# Integrated metabolomic, transcriptomic and computational analyses reveal geographical variation and potential anti-osteoarthritis bioactive compounds in *Arenga pinnata* pith

**DOI:** 10.3389/fpls.2026.1974434

**Published:** 2026-10-21

**Authors:** XiangXin Xie, Marini Othman, HongYun Chen, XiMing Li, JunYan Li, JinMei Xie, ZhiHao Liu

**Affiliations:** 1 Faculty of Information Engineering, Nantong Institute of Technology, Nantong, China; 2 Faculty of Data Science and Information Technology, INTI International University, Nilai, Malaysia; 3 Department of Information Engineering, Heilongjiang Communications Polytechnic, Herbin, China; 4 Faculty of Artificial Intelligence, Dongguan City University, Dongguan, China; 5 Faculty of Intelligent Engineering, Guangxi City Vocational University, Chongzuo, China

**Keywords:** *Arenga pinnata*, flavonoids, geographical variation, metabolomics, osteoarthritis, transcriptomics, human health

## Abstract

*Arenga pinnata* pith is an underexplored plant resource whose chemical and transcriptional characteristics may vary substantially among geographical populations. Here, we performed a comparative multi-omics analysis of pith samples collected from Hainan, Jiangxi, and Taiwan in China, as well as Thailand and Indonesia, using untargeted metabolomics and transcriptome sequencing. Metabolomic profiling detected 1,707 metabolite features spanning major classes including amino acids and derivatives, flavonoids, phenolic acids, alkaloids, and lipids. Clear geographical differences were observed in both metabolite composition and accumulation patterns. Taiwan samples showed the greatest overall metabolite diversity and abundance, whereas pathway enrichment highlighted recurrent differences in flavonoid and phenylpropanoid biosynthesis, amino-acid metabolism, lipid metabolism, central carbon metabolism, and glutathione metabolism. Transcriptomic profiling likewise revealed pronounced population-level differentiation. Hainan and Jiangxi exhibited comparatively similar expression patterns, while the Thailand population showed the strongest transcriptional divergence, including 10,720 and 10,284 differentially expressed genes relative to Hainan and Taiwan, respectively. Flavonoid-related variation observed in the metabolomic dataset further motivated a secondary exploratory analysis in which quercetin and naringenin were used as representative flavonoid scaffolds to investigate possible osteoarthritis (OA)-related molecular associations. Network analysis identified AKT1, MMP9, PTGS2, PPARG, MMP2, and MMP13 as highly connected candidate targets. Independent analysis of the human OA dataset GSE114007 identified DDIT3, KLF10, CFI, and MAFF as candidate OA-associated biomarkers, and molecular docking predicted favorable interactions of quercetin and naringenin with AKT1 and MMP9. These OA-related results are computational and hypothesis-generating and should not be interpreted as evidence of anti-OA activity of *A. pinnata* pith. Collectively, this study defines substantial geographical variation in the metabolomic and transcriptomic profiles of *A. pinnata* pith and provides a comparative framework for resource evaluation, chemical characterization, and future experimental investigation of selected flavonoid-related constituents.

## 1 Introduction

As an important tropical plant resource of the family Arecaceae, *Arenga pinnata* is widely distributed in tropical and subtropical regions and possesses considerable ecological, economic, and potential medicinal value. Historically, *A. pinnata* has primarily been utilized as a tropical economic crop, while its pith has a long-standing history of use as both a food source and a traditional folk medicine (Sarkar et al., 2022). With the continued development of research on natural medicines and functional plants, tropical plants rich in bioactive metabolites have increasingly become important sources for natural product development. The diverse secondary metabolites present in plants not only participate in plant growth, development, and environmental adaptation but are also closely associated with various physiological activities, including antioxidant, anti-inflammatory, and immunomodulatory effects (Xiao et al., 2025). Therefore, systematic investigation of the chemical constituents of *A. pinnata* pith may not only help elucidate its potential medicinal value but also provide a theoretical basis for resource development and functional utilization.

*A. pinnata* is mainly distributed in southern China and Southeast Asia (Harahap et al., 2025). Considerable differences in temperature, precipitation, light exposure, altitude, soil composition, and other ecological conditions exist among different geographical origins. These environmental factors can directly influence metabolic pathways and the regulation of gene expression in plants, thereby resulting in geographical variation in metabolite composition and accumulation levels. The biosynthesis of plant secondary metabolites is governed not only by genetic background but also by transcriptional regulation associated with environmental adaptation. Consequently, *A. pinnata* populations from different regions may develop geographically distinctive metabolic patterns and gene-expression profiles. Systematic comparison of the chemical composition and molecular regulatory differences among *A. pinnata* populations from different geographical origins is therefore important for elucidating the mechanisms underlying bioactive compound formation and identifying superior plant resources (Awal et al., 2025).

Metabolomics can directly reflect changes in the diversity and abundance of small-molecule metabolites in plants, thereby revealing differences in chemical composition among different plant materials (Shen et al., 2023). Transcriptomics, by contrast, enables the characterization of expression changes in key structural genes, regulatory genes, and transcription factors involved in metabolic pathways, thus providing further insight into the molecular regulatory mechanisms underlying metabolite accumulation. The biosynthesis of bioactive plant metabolites generally depends on the coordinated action of a series of metabolic enzymes, while their accumulation is regulated by the expression levels of genes encoding key enzymes within the corresponding metabolic pathways. Therefore, integrating metabolomic and transcriptomic analyses enables a joint investigation extending from the “metabolic phenotype” to the “gene regulatory network,” thereby providing a more comprehensive understanding of the biosynthetic mechanisms of bioactive plant constituents (Li and Gaquerel, 2021).

Osteoarthritis (OA) is a chronic degenerative joint disorder characterized by cartilage degradation, extracellular matrix remodeling, and inflammatory responses. Plant-derived flavonoids have attracted attention as potential modulators of inflammation, oxidative stress, and matrix homeostasis, providing a rationale for computational evaluation of selected flavonoid-related compounds in OA-associated molecular networks. However, the occurrence of such compounds in a plant metabolomic profile does not by itself demonstrate pharmacological activity. Therefore, in the present study, OA-related network analysis was used only as a secondary, hypothesis-generating framework to explore possible molecular associations of selected compounds, rather than as evidence that *A. pinnata* pith has experimentally demonstrated anti-OA effects (Chen et al., 2017).

Accordingly, the primary objective of this study was to determine whether *A. pinnata* pith from different geographical origins exhibits distinct and reproducible metabolomic and transcriptomic profiles, and to identify the major metabolic and biological pathways associated with this geographical differentiation. Pith samples collected from Hainan, Jiangxi, and Taiwan in China, as well as Thailand and Indonesia, were therefore characterized using untargeted metabolomics and transcriptome sequencing. We first compared metabolite composition, differential accumulation patterns, gene-expression profiles, and enriched pathways among the five geographical groups. As a secondary and exploratory analysis, selected flavonoid-related compounds were further evaluated using network pharmacology and molecular docking to generate hypotheses regarding possible osteoarthritis-related molecular interactions. In parallel, an independent human OA transcriptomic dataset was analyzed using machine-learning approaches to identify candidate disease-associated genes. These computational analyses were not intended to demonstrate anti-OA activity of *A. pinnata* pith, but rather to provide testable hypotheses for subsequent pharmacological validation. Overall, this study focuses primarily on geographical variation in *A. pinnata* pith and provides a molecular basis for germplasm evaluation, chemical quality assessment, and future investigation of its bioactive constituents.

## 2 Methods

### 2.1 Plant material collection and preparation

Fresh *A. pinnata* pith samples were obtained from five geographical origins: Sanya, Hainan Province, China (HN); Ganzhou, Jiangxi Province, China (JX); Chiayi, Taiwan, China (TW); Trang, Thailand (TG); and Bogor, West Java, Indonesia (YN). Three independent biological replicates were included for each geographical group. The plant materials were authenticated as *A. pinnata* (Wurmb) Merr. by Prof. Marini Othman. Voucher specimens were deposited in the Herbarium of INTI International University under accession numbers HN2025001, JX2025001, TW2025001, TG2025001, and YN2025001, respectively. All collection procedures complied with applicable local laws and regulations.

### 2.2 Untargeted metabolomics

#### 2.2.1 Sample preparation

Approximately 120 mg of fresh *A. pinnata* pith was ground to a fine powder in liquid nitrogen and extracted with 1.2 mL of ice-cold methanol/acetonitrile/water (3:2:1, v/v/v). After vortexing for 2 min, the samples were sonicated in an ice-water bath for 15 min and then flash-frozen in liquid nitrogen for 2 min. Following centrifugation at 13,000 × g for 20 min at 4 °C, the supernatants were collected and evaporated to dryness under a gentle stream of nitrogen. The dried residues were reconstituted in 150 μL of acetonitrile/water (2:1, v/v), vortexed for 1 min, sonicated in an ice bath for 5 min, and centrifuged again at 13,000 × g for 10 min at 4 °C. The resulting supernatants were transferred to LC–MS vials for subsequent metabolomic analysis (Li et al., 2026).

#### 2.2.2 UHPLC-QTOF-MS analysis

An ACQUITY UPLC BEH Amide column (100 mm × 2.1 mm, 1.7 μm; Waters) was maintained at 25 °C and used for hydrophilic interaction liquid chromatographic separation. The aqueous mobile phase contained 25 mM ammonium acetate and 25 mM ammonium hydroxide (solvent A), whereas acetonitrile served as solvent B. Samples were eluted at a constant flow rate of 0.30 mL/min using the gradient program presented in **Table 1**, and the injection volume was 2 μL. To minimize instrumental drift and injection-order effects, all samples were analyzed in a randomized sequence. The chromatographic system was coupled to an AB Sciex TripleTOF 5600+ quadrupole time-of-flight mass spectrometer for metabolite detection (Li et al., 2026).

**Table 1:** Gradient elution program for UPLC analysis.

Time (min)	Mobile phase B (%)
0-1	95
1-14	95 → 65
14-16	65 → 40
16-18	40
18-18.1	40 → 95
18.1-23	95

Data were acquired separately under positive- and negative-ion electrospray ionization conditions. The spray voltages were set to +5,500 and −5,500 V, respectively, and the source temperature was maintained at 600 °C. Gas1 and Gas2 were both supplied at 60 psi, while the curtain gas pressure was set to 30 psi. Full-scan mass spectra were recorded over an *m/z* range of 60–1,000 with an accumulation time of 0.20 s per scan. Fragment-ion spectra were acquired in information-dependent acquisition mode over an *m/z* range of 25–1,000, with an accumulation time of 0.05 s per MS/MS scan. Precursor ions were fragmented using a collision energy of 35 ± 15 eV, and the declustering potential was set to +60 V in positive-ion mode and −60 V in negative-ion mode. During each acquisition cycle, up to 10 precursor ions were selected for fragmentation. Isotopic ions within a 4 Da mass window were excluded from precursor selection to reduce redundant fragmentation and improve spectral quality.

#### 2.2.3 Data processing and metabolite annotation

Raw UHPLC-QTOF-MS data were converted to an open data format using ProteoWizard MSConvert and subsequently processed using MS-DIAL (version 4.92) for peak detection, deconvolution, retention-time alignment, and feature extraction (Kessner et al., 2008; Tsugawa et al., 2015). Features detected in fewer than 50% of the biological replicates within any experimental group were excluded from subsequent analysis.

Metabolite annotation was performed by matching the accurate precursor mass and MS/MS fragmentation spectra against publicly available spectral and metabolite databases, including the Human Metabolome Database (HMDB), MassBank, the MassBank of North America (MoNA), METLIN, and the Kyoto Encyclopedia of Genes and Genomes (KEGG) (Horai et al., 2010; Minoru Kanehisa et al., 2023; Smith et al., 2005; Wishart et al., 2022). Candidate annotations were accepted when the precursor-ion mass error was within 10 ppm and the major MS/MS fragment ions were consistent with the reference spectra, with a spectral similarity score of ≥80% where library spectra were available. Molecular formula, isotope distribution, and fragmentation characteristics were additionally considered when assigning candidate structures.

#### 2.2.4 Statistical analysis of metabolomic data

Metabolite abundance data were log2-transformed and normalized prior to statistical analysis. Differential metabolites between geographical groups were screened using a combination of fold change and statistical significance. Metabolites with an absolute log2 fold change (|log2FC|) ≥ 1 and a nominal *P* value < 0.05 were considered differentially accumulated. To control for multiple comparisons, *P* values were adjusted using the Benjamini–Hochberg false discovery rate (FDR) procedure, and metabolites with FDR < 0.05 were retained for subsequent interpretation (Benjamini and Hochberg, 1995). Volcano plots and hierarchical-clustering heatmaps were generated based on the resulting differential metabolite sets.

### 2.3 Transcriptome sequencing

#### 2.3.1 RNA extraction and library construction

Approximately 100 mg of fresh *A. pinnata* pith was ground to a fine powder in liquid nitrogen for total RNA extraction. Total RNA was extracted using the RNeasy Plant Mini Kit (QIAGEN, Hilden, Germany) with on-column DNase I digestion to remove genomic DNA. RNA quantity and purity were assessed using a NanoDrop 2000 spectrophotometer (Thermo Fisher Scientific, Waltham, MA, USA), and RNA integrity was evaluated using an Agilent 2100 Bioanalyzer (Agilent Technologies, Santa Clara, CA, USA). Only samples with A260/A280 ratios of approximately 2.0 and an RNA Integrity Number (RIN) > 8.0 were used for library preparation (Jiang et al., 2024). Poly(A)+ mRNA was enriched using oligo(dT) magnetic beads, fragmented, and reverse transcribed into cDNA. Paired-end libraries were prepared using the TruSeq RNA Library Prep Kit (Illumina, San Diego, CA, USA) and sequenced on an Illumina NovaSeq 6000 platform (Illumina, San Diego, CA, USA) to generate 150-bp paired-end reads.

#### 2.3.2 Functional annotation and quantification

Assembled unigenes were annotated by BLASTx searches (*E*-value < 1e-5) against the NCBI non-redundant protein (NR) database (Sayers et al., 2022), Swiss-Prot database (Boutet et al., 2016) and KEGG protein database(M. Kanehisa et al., 2023). Gene Ontology (GO) terms were assigned using Blast2GO (Götz et al., 2008) based on NR hits, and protein domains were identified using the Pfam database (Mistry et al., 2021). Gene expression levels were quantified by aligning clean reads back to assembled transcripts using RSEM v.1.3.3 (Li and Dewey, 2011). Transcript abundance for each unigene was expressed as transcripts per million (TPM).

#### 2.3.3 Differential gene expression analysis

Differential gene-expression analysis among geographical groups was performed on the normalized transcript abundance data. Genes with |log2 fold change| ≥ 1 and an FDR-adjusted *P* value < 0.05 were considered differentially expressed genes (DEGs). Multiple-testing correction was performed using the Benjamini–Hochberg procedure (Benjamini and Hochberg, 1995). Genes with positive log2FC values were classified as upregulated, whereas those with negative values were classified as downregulated. The resulting DEG sets were used for volcano-plot visualization and subsequent functional enrichment analyses.

### 2.4 Construction of the target set related to osteoarthritis

Genes related to OA were retrieved from the GeneCards (https://www.genecards.org/) and DisGeNet (https://www.disgenet.org/) databases using “osteoarthritis” as the keyword to search for the disease targets. Gene names were standardized using the UniProt database (https://www.uniprot.org/) (Bateman et al., 2025). To ensure the reliability of the data, targets with a high correlation value (≥0.15) were selected.

### 2.5 PPI network construction and analysis

The candidate quercetin–OA and naringenin–OA target sets obtained from the network pharmacology analysis were separately submitted to the STRING database for PPI analysis, with *Homo sapiens* selected as the organism (Szklarczyk et al., 2023). A high-confidence interaction score threshold of 0.900 was applied. The resulting interaction networks were imported into Cytoscape v3.10.0 for visualization and topological analysis (Shannon et al., 2003). Node degree was calculated to characterize network connectivity and to identify highly connected candidate targets. It should be emphasized that the proteins analyzed in the PPI networks are predicted human OA-related targets of the selected compounds and are independent of the genes identified from the *A. pinnata* transcriptomic dataset.

### 2.6 Identification of key therapeutic targets of OA based on machine learning

The publicly available human gene-expression dataset GSE114007 was obtained from the Gene Expression Omnibus (GEO) database. This dataset comprises 20 osteoarthritis (OA) samples and 18 control samples and was used exclusively for secondary computational analysis in the present study. No new human participants were recruited and no human biological specimens were collected by the authors. Because of the relatively small sample size, an independent train-test split was not used, as this would further reduce the number of samples available for model development and evaluation. Instead, model performance was assessed using repeated stratified five-fold cross-validation. In each repetition, samples were randomly divided into five folds while preserving the relative proportions of OA and control samples. Four folds were used for model training and the remaining fold was used for validation, and this procedure was repeated until each fold had served as the validation set.

Statistical analyses were performed using R software (version 4.2.2). All machine-learning and co-expression analyses were performed using established, publicly available R packages and published algorithms; no custom analysis software or novel algorithm was developed in this study. Weighted Gene Co-expression Network Analysis (WGCNA) was carried out using the WGCNA R package (version 1.72-1) (Langfelder and Horvath, 2008), in which the adjacency matrix was converted into a topological overlap matrix (TOM), and the topological overlap dissimilarity measure (1-TOM) was used for hierarchical clustering analysis. Gene modules were subsequently identified using the dynamic tree cut method. To further screen for key genes, least absolute shrinkage and selection operator (LASSO) regression analysis was conducted using the glmnet package (version 4.1-8) with cross-validation (Friedman et al., 2010). In parallel, random forest (RF) analysis was performed using the randomForest package (version 4.7-1.1) (Liaw and Wiener, 2002). Support vector machine-recursive feature elimination (SVM-RFE) was implemented using the e1071 package (version 1.7-14), following the established SVM-RFE framework (Guyon et al., 2002; Meyer et al., 2019).

### 2.7 Molecular docking

Quercetin and naringenin were selected as representative flavonoid compounds for molecular docking with the shared hub targets AKT1 and MMP9 identified from the preceding PPI network analyses. The three-dimensional structures of quercetin and naringenin were obtained in SDF format from the PubChem database, and the crystal structures of human AKT1 (PDB ID: 3O96) and MMP9 (PDB ID: 1GKC) were obtained from the RCSB Protein Data Bank. The AKT1 structure represents a human AKT1 complex containing an experimentally resolved allosteric ligand, whereas the MMP9 structure contains the catalytic domain of human MMP9 in complex with an inhibitor.

Prior to docking, the receptor structures were prepared by removing crystallographic water molecules and the co-crystallized ligands, while retaining the catalytically relevant Zn²^+^ ion in MMP9. Polar hydrogen atoms were added and appropriate atomic charges were assigned using AutoDockTools (version 1.5.7){Morris, 2009 #62}. Ligand structures were prepared by adding hydrogen atoms, assigning charges, and defining rotatable bonds. A semi-flexible docking strategy was adopted, in which the receptor proteins were treated as rigid, whereas quercetin and naringenin were allowed to rotate around their rotatable bonds. Molecular docking was subsequently performed using AutoDock Vina {Trott, 2010 #64}.

The docking search spaces were defined according to the binding regions occupied by the corresponding co-crystallized ligands. For AKT1, the grid box was centered at X = 8.37, Y = −6.83, and Z = 12.62 Å, with dimensions of 28 × 28 × 28 Å, thereby covering the allosteric ligand-binding region at the interface of the PH and kinase domains. For MMP9, the grid box was centered at X = 65.84, Y = 30.71, and Z = 117.75 Å, with dimensions of 24 × 24 × 24 Å, encompassing the catalytic pocket and the catalytic Zn²^+^-containing region. The grid dimensions were selected to fully cover the corresponding binding pockets and allow adequate conformational exploration of the ligands during docking.

For docking-protocol validation, the native co-crystallized ligands of AKT1 and MMP9 were used as reference ligands for re-docking into their corresponding binding pockets. The agreement between the re-docked pose and the crystallographic conformation was evaluated using the root-mean-square deviation (RMSD), with an RMSD of <2.0 Å considered indicative of acceptable reproduction of the experimentally observed binding mode. The resulting protein–ligand complexes were visualized and analyzed using PyMOL (version 2.5.0), with particular attention to hydrogen bonding, hydrophobic interactions, aromatic interactions, and interactions involving key catalytic or binding-site residues.

The predicted binding energy was used for comparative evaluation of the protein–ligand interactions, with more negative binding-energy values indicating more favorable predicted structural interactions under the applied docking conditions. The docking results were interpreted as exploratory computational predictions of potential compound–target interactions rather than as direct experimental confirmation of molecular binding, target activation or inhibition, or therapeutic efficacy.

## 3 Results

### 3.1 Metabolomic analysis of *A. pinnata* pith

#### 3.1.1 Overall metabolomic profile

Metabolic profiling of *A. pinnata* pith from five geographical origins identified 1,707 metabolites, including 334 amino acids and derivatives, 327 flavonoids, 238 other compounds, 216 phenolic acids, 132 alkaloids, 125 lipids, 100 lignans and coumarins, 85 organic acids, 62 nucleotides and derivatives, 53 terpenoids, 19 tannins, and 16 quinones. Thus, the metabolite profile comprised diverse primary and secondary metabolites, particularly amino acids, flavonoids, and phenolic acids (**Supplementary Table S1**).

The Taiwan group contained the largest number of metabolites (1,677) and showed the highest total abundance signal. Amino acids and derivatives (29.2%) and flavonoids (19.5%) were the major contributors, followed by other compounds (11.4%), alkaloids (11.0%), phenolic acids (7.4%), organic acids (6.1%), and lipids (5.7%). The three biological replicates contained 1,663, 1,665, and 1,661 metabolites, respectively, indicating excellent reproducibility.

The Hainan group contained 1,661 metabolites and was dominated by amino acids and derivatives (29.8%), followed by alkaloids (12.4%), other compounds (12.3%), lipids (11.2%), organic acids (11.0%), flavonoids (9.5%), and phenolic acids (6.5%). The replicate metabolite counts were 1,647, 1,648, and 1,646. By contrast, the Jiangxi group contained the fewest metabolites (1,520), although flavonoids remained highly diverse, with 305 compounds detected. Its major abundance classes were amino acids and derivatives (20.2%), other compounds (19.3%), and flavonoids (16.2%). The three replicates contained 1,490, 1,492, and 1,498 metabolites, showing high within-group consistency.

The Thailand group contained 1,655 metabolites and was characterized by relatively high proportions of amino acids and derivatives (25.7%), other compounds (17.4%), alkaloids (13.3%), and flavonoids (12.3%). The three replicates contained 1,638, 1,644, and 1,638 metabolites. The Indonesia group contained 1,672 metabolites and exhibited a relatively balanced composition, with amino acids and derivatives accounting for 23.5%, flavonoids for 15.1%, other compounds for 14.5%, alkaloids for 11.0%, and lipids for 10.2%. It also contained the highest number of quinones among the five groups. The three replicates contained 1,657, 1,658, and 1,656 metabolites.

Overall, the Taiwan group exhibited the greatest metabolite diversity and abundance, the Hainan group was enriched in amino acids, organic acids, lipids, and alkaloids, and the Jiangxi group showed lower overall diversity but a relatively high flavonoid proportion. The Thailand group was characterized by higher proportions of alkaloids and other compounds, whereas the Indonesia group displayed a broad and balanced metabolite profile. These results demonstrate clear geographical variation in the metabolite composition of *A. pinnata* pith.

#### 3.1.2 Differential metabolomic analysis

A total of 916 differential metabolites were identified between the Jiangxi and Hainan *A. pinnata* pith samples. Relative to Hainan, 319 metabolites were upregulated and 597 were downregulated in Jiangxi, indicating that most differential metabolites accumulated at higher levels in Hainan. Similarly, among the 589 differential metabolites identified between Thailand and Hainan, 220 were upregulated and 369 were downregulated in Thailand. In contrast, Taiwan showed a pronounced overall enrichment relative to Hainan, with 481 of 698 differential metabolites upregulated and 217 downregulated (The complete differential metabolite datasets, including the corresponding statistical information, are available through the public data link provided in the Data Availability statement).

In the Jiangxi-Hainan comparison, differential metabolites were mainly classified as amino acids and derivatives, flavonoids, phenolic acids, and other compounds. Amino acids and derivatives were the largest class, with 29 upregulated and 194 downregulated metabolites, indicating greater accumulation in Hainan. By contrast, flavonoids were predominantly enriched in Jiangxi, with 113 upregulated and 56 downregulated metabolites. Phenolic acids and other compounds were mainly enriched in Hainan, whereas lipids, particularly lysophospholipids, showed slight enrichment in Jiangxi. Lignans and coumarins, organic acids, and nucleotides and their derivatives were also predominantly lower in Jiangxi (**Figures 1A**, **2A**).

**Figure 1:**
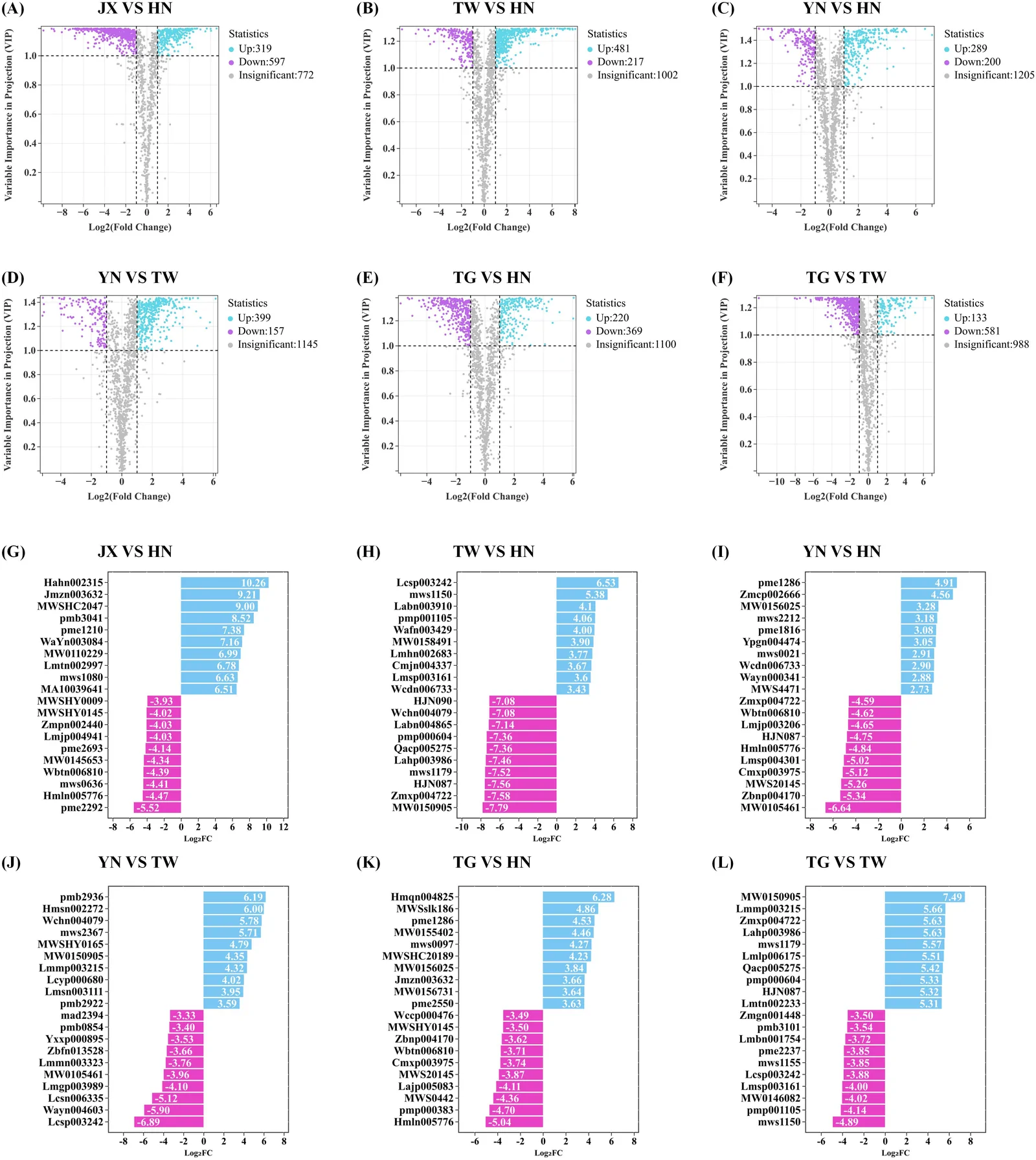
Differential metabolite analysis of *A. pinnata* pith from different geographical origins [**(A)** Volcano plot of differential metabolites in JX vs. HN; **(B)** volcano plot of differential metabolites in TW vs. HN; **(C)** volcano plot of differential metabolites in YN vs. HN; **(D)** volcano plot of differential metabolites in YN vs. TW; **(E)** volcano plot of differential metabolites in TG vs. HN; **(F)** volcano plot of differential metabolites in TG vs. TW; **(G)** bar plot of differential metabolites in JX vs. HN; **(H)** bar plot of differential metabolites in TW vs. HN; **(I)** bar plot of differential metabolites in YN vs. HN; **(J)** bar plot of differential metabolites in YN vs. TW; **(K)** bar plot of differential metabolites in TG vs. HN; and **(L)** bar plot of differential metabolites in TG vs. TW].

**Figure 2:**
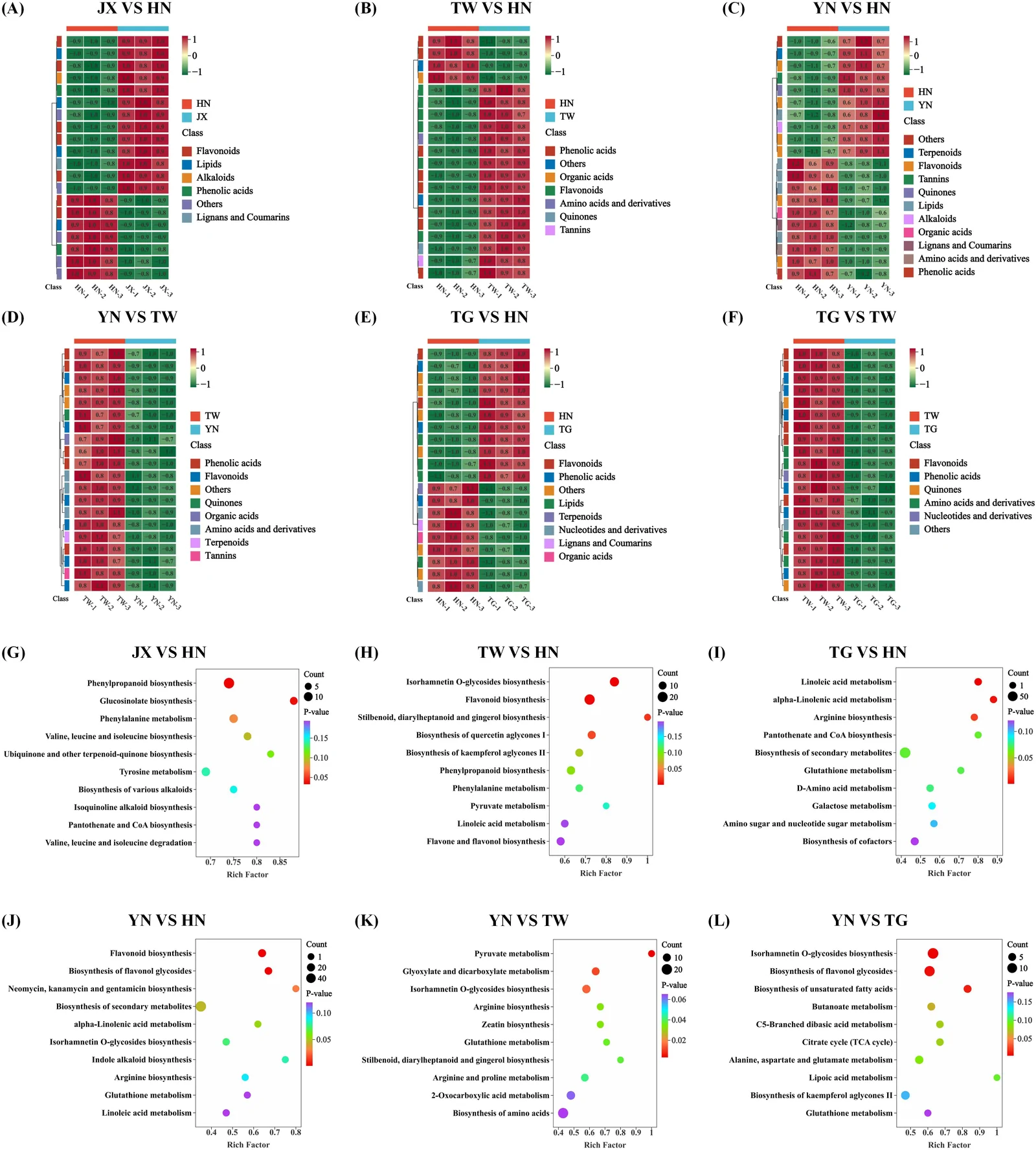
Heatmaps and KEGG analysis of differential metabolites in *A. pinnata* pith from different geographical origins. [**(A)** Heatmap of differential metabolites in JX vs. HN; **(B)** heatmap of differential metabolites in TW vs. HN; **(C)** heatmap of differential metabolites in YN vs. HN; **(D)** heatmap of differential metabolites in YN vs. TW; **(E)** heatmap of differential metabolites in TG vs. HN; **(F)** heatmap of differential metabolites in TG vs. TW; **(G)** KEGG analysis of differential metabolites in JX vs. HN; **(H)** KEGG analysis of differential metabolites in TW vs. HN; **(I)** KEGG analysis of differential metabolites in YN vs. HN; **(J)** KEGG analysis of differential metabolites in YN vs. TW; **(K)** KEGG analysis of differential metabolites in TG vs. HN; **(L)** KEGG analysis of differential metabolites in TG vs. TW].

Representative metabolites enriched in Jiangxi included Flavogallonic Acid Dilactone, Cimicifugic acid E-Diglucose, Isoorientin-7-O-glucoside, and Putrescine, whereas syringaresinol glycosides, Manglieside D, and Tricin-7-O-saccharic acid were reduced. Overall, Jiangxi was characterized by higher levels of flavonoids and selected lipids but lower levels of amino acids and derivatives, lignans, organic acids, nucleotides and their derivatives, and certain phenolic acids (**Figure 1G**).

The differential metabolites between the Thailand and Hainan groups were mainly flavonoids, amino acids and derivatives, other compounds, phenolic acids, lipids, lignans, and coumarins. Flavonoids were the largest class, with 60 upregulated and 40 downregulated metabolites, indicating greater accumulation of selected flavonoids in Thailand. In contrast, amino acids and derivatives, phenolic acids, lipids, lignans, and coumarins were predominantly decreased, suggesting higher accumulation in Hainan. Free fatty acids were mainly enriched in Hainan, whereas lysophospholipids, including lysophosphatidylcholines, were more abundant in Thailand (**Figures 1E**, **2E**).

Representative metabolites enriched in Thailand included Cimicifugic acid E-Diglucose, Acacetin-6-C-glucoside, Capsianoside IV, and 1,4-Dihydro-1-Methyl-4-oxo-3-pyridinecarboxamide, whereas Rabdosia acid A, N-Acetyl-L-Asparagine, Piceatannol, Disinapoyl glucoside, and Dihydroferulic acid glucoside were reduced. Overall, Thailand showed higher levels of flavonoids, selected carbohydrates, alkaloids, and lysophospholipids, but lower levels of amino acids and derivatives, lignans, free fatty acids, organic acids, and selected phenolic acids than Hainan (**Figure 1K**).

The differential metabolites between the Taiwan and Hainan *A. pinnata* pith samples were mainly classified as flavonoids, phenolic acids, amino acids and their derivatives, and other compounds. Flavonoids constituted the largest category, with 208 differential metabolites accounting for 29.8% of the total. Among them, 178 were upregulated and 30 were downregulated, showing a pronounced overall increase and representing the most prominent metabolite class enriched in the Taiwan samples relative to the Hainan samples. Phenolic acids comprised 107 metabolites, accounting for 15.3%, of which 69 were upregulated and 38 were downregulated. This overall upregulation indicated that many phenolic acids accumulated to higher levels in the Taiwan samples than in the Hainan samples. Amino acids and their derivatives comprised 101 metabolites, accounting for 14.5%, including 79 upregulated and 22 downregulated compounds. This clear upregulation contrasted with the predominantly downregulated pattern observed for amino acids and their derivatives in the Jiangxi-versus-Hainan and Thailand-versus-Hainan comparisons, indicating a distinctive capacity for the accumulation of amino acid-related metabolites in the Taiwan samples. Other compounds comprised 81 metabolites, accounting for 11.6%, of which 46 were upregulated and 35 were downregulated, showing a slight overall increase.

In addition, 48 differential lipids were identified, including 20 upregulated and 28 downregulated metabolites, indicating a slight overall enrichment of lipids in the Hainan samples, particularly free fatty acids. Among the 37 differential organic acids, 11 were upregulated and 26 were downregulated, indicating that organic acids also accumulated predominantly in the Hainan samples. By contrast, alkaloids, terpenoids, nucleotides, and their derivatives were predominantly upregulated in the Taiwan samples (**Figures 1B**, **2B**).

Representative metabolites upregulated in the Taiwan samples relative to the Hainan samples included Padmakastin, 3,4,5-Trihydroxy-6-[4-(4-methyl-3-oxopentyl)phenoxy]oxane-2-carboxylic acid, His-Gln-Thr, Thunberginol G 3′-Glucoside, and Naringenin-4′-O-glucoside. Representative downregulated metabolites included Resveratrol, Dihydrozeatin ribonucleoside, Pro-Thr-His, Quercetin-3,7-Di-O-glucoside, and Piceatannol. Overall, the Taiwan samples exhibited the most pronounced global upregulation pattern relative to the Hainan samples, with particularly high accumulation of flavonoids, phenolic acids, amino acids and their derivatives, alkaloids, terpenoids, nucleotides, and their derivatives. In contrast, the Hainan samples showed relative enrichment of organic acids, free fatty acids, certain lignans, and a small number of flavonoids (**Figure 1H**).

Marked metabolic differences were observed between the Indonesian *A. pinnata* pith samples and those from the other geographical origins, although the directions of change varied among comparisons. A total of 489 differential metabolites were identified between the Indonesian and Hainan samples. Relative to the Hainan group, 289 metabolites were upregulated in the Indonesian group, accounting for 59.1%, whereas 200 were downregulated, accounting for 40.9%. The overall predominance of upregulated metabolites indicated that most differential metabolites were present at higher levels in the Indonesian samples. A total of 556 differential metabolites were identified between the Indonesian and Thailand groups, of which 399 were upregulated in the Indonesian samples, accounting for 71.8%, and 157 were downregulated, accounting for 28.2%. This more pronounced overall upregulation pattern suggested that the Indonesian samples had a broader advantage in metabolite accumulation than the Thailand samples. By contrast, 556 differential metabolites were identified between the Indonesian and Taiwan groups. Among them, 399 were upregulated in the Indonesian samples, accounting for 71.8%, whereas 157 were downregulated, accounting for 28.2%. The predominance of upregulated metabolites indicated that a larger proportion of differential metabolites accumulated at higher levels in the Indonesian samples than in the Taiwan samples (**Figure 1D**).

In the Indonesia-Hainan comparison, differential metabolites were mainly classified as flavonoids, phenolic acids, amino acids and derivatives, lipids, and other compounds. Flavonoids were the predominant class, with 109 upregulated and 25 downregulated metabolites, indicating marked enrichment in the Indonesian samples. Phenolic acids also showed an overall increase, whereas amino acids and derivatives were mainly reduced, suggesting greater accumulation in Hainan. Lipids showed a relatively balanced pattern, while other compounds were generally more abundant in Indonesia (**Figures 1C**, **2C**). Representative metabolites enriched in Indonesia included Cimicifugic acid E-Diglucose, Ala-Cys-Gly, Eriodictyol-3′-O-glucoside, Eriodictyol-7-O-glucoside, and LysoPE 18:3, whereas Dihydroferulic Acid, S-(5′-Adenosy)-L-homocysteine, N-alanicacidalkyl-alanine, L-Saccharopine, and Leu-Thr-Asp were reduced (**Figure 1I**).

In the Taiwan-Thailand comparison, flavonoids, phenolic acids, other compounds, amino acids and derivatives, lipids, lignans, and coumarins were predominantly enriched in Taiwan. The strongest increases were observed for flavonoids, lipids, and lignans and coumarins, indicating a broad metabolite accumulation advantage in the Taiwan samples (**Figures 1F**, **2F**). Representative upregulated metabolites included Disinapoyl glucoside, Nodakenin, 3,4,5-Trihydroxy-6-[4-(4-methyl-3-oxopentyl)phenoxy]oxane-2-carboxylic acid, and Quercetin-3-O-sophoroside (Baimaside). Chrysoeriol-5,7-di-O-glucoside, 2-Picoline/2-Methylpyridine, oxyphyllone F, and Ribitol were downregulated (**Figure 1L**).

The differential metabolites between the Indonesian and Taiwan samples were mainly distributed among flavonoids, amino acids and their derivatives, phenolic acids, other compounds, and lipids. Overall, the YN vs. TW comparison showed a predominance of upregulated metabolites in the Indonesian samples, consistent with the volcano-plot statistics shown in **Figure 1D**. Representative differential metabolites included Glucosylviscumneoside III, Quercetin-3-O-Sambubioside-5-O-Glucoside, 6-Hydroxyrumicin-8-O-D-glucoside, Quercetin-4′-O-glucuronide, Dihydrozeatin ribonucleoside, Disinapoyl glucoside, Juglanoside D, Putrescine, Demethyl coniferin, and L-Homomethionine (**Figure 1J**).

Representative metabolites upregulated in the Indonesian samples relative to the Taiwan samples included Glucosylviscumneoside III, Quercetin-3-O-Sambubioside-5-O-Glucoside, 6-Hydroxyrumicin-8-O-D-glucoside, Quercetin-4′-O-glucuronide, and Dihydrozeatin ribonucleoside. Representative downregulated metabolites included Disinapoyl glucoside, Juglanoside D, Putrescine, Demethyl coniferin, and L-Homomethionine.

#### 3.1.3 KEGG analysis of differential metabolites

KEGG pathway enrichment analysis was performed for the differential metabolites identified in the six pairwise comparisons. The results showed that these metabolites were mainly involved in the biosynthesis of plant secondary metabolites, flavonoids and flavonol glycosides, phenylpropanoid biosynthesis, fatty acid metabolism, amino acid metabolism, central carbon metabolism, carbohydrate metabolism, and glutathione metabolism. Although the composition of enriched pathways differed among the comparison groups, the overall results indicated the coordinated involvement of secondary metabolism, lipid metabolism, amino acid metabolism, and energy metabolism.

Among the comparisons with HN, the differential metabolites in JX vs. HN were mainly enriched in Phenylpropanoid biosynthesis, Glucosinolate biosynthesis, Phenylalanine metabolism, Valine, leucine and isoleucine biosynthesis, Tyrosine metabolism, and alkaloid-related biosynthetic pathways. The enrichment profile was primarily characterized by phenylpropanoid metabolism, glucosinolate metabolism, aromatic amino acid metabolism, and branched-chain amino acid metabolism (**Figure 2G**). In TW vs. HN, the differential metabolites were mainly enriched in Isorhamnetin O-glycosides biosynthesis, Flavonoid biosynthesis, Biosynthesis of quercetin aglycones I, Biosynthesis of kaempferol aglycones II, Phenylpropanoid biosynthesis, Phenylalanine metabolism, and Flavone and flavonol biosynthesis. Overall, the enriched pathways were concentrated in flavonoid and flavonol metabolism and the upstream phenylpropanoid pathway (**Figure 2H**). In TG vs. HN, the differential metabolites were mainly enriched in Linoleic acid metabolism, alpha-Linolenic acid metabolism, Arginine biosynthesis, Pantothenate and CoA biosynthesis, Biosynthesis of secondary metabolites, Glutathione metabolism, Galactose metabolism, and Amino sugar and nucleotide sugar metabolism. These pathways were primarily associated with unsaturated fatty acid metabolism, arginine metabolism, glutathione metabolism, carbohydrate metabolism, and cofactor biosynthesis (**Figure 2I**). In YN vs. HN, the differential metabolites were mainly enriched in Flavonoid biosynthesis, Biosynthesis of flavonol glycosides, Biosynthesis of secondary metabolites, alpha-Linolenic acid metabolism, Isorhamnetin O-glycosides biosynthesis, Indole alkaloid biosynthesis, Arginine biosynthesis, Glutathione metabolism, and Linoleic acid metabolism. This comparison showed the simultaneous enrichment of flavonoid and flavonol glycoside pathways, secondary-metabolite biosynthesis, and fatty acid metabolism (**Figure 2J**).

Among the comparisons between YN and the other groups, the differential metabolites in YN vs. TW were mainly enriched in Pyruvate metabolism, Glyoxylate and dicarboxylate metabolism, Isorhamnetin O-glycosides biosynthesis, Arginine biosynthesis, Zeatin biosynthesis, Glutathione metabolism, Arginine and proline metabolism, 2-Oxocarboxylic acid metabolism, and Biosynthesis of amino acids. The enriched pathways were mainly associated with central carbon metabolism, organic acid metabolism, amino acid metabolism, plant hormone-related metabolism, and glutathione metabolism (**Figure 2K**). In YN vs. TG, the differential metabolites were mainly enriched in Isorhamnetin O-glycosides biosynthesis, Biosynthesis of flavonol glycosides, Biosynthesis of unsaturated fatty acids, Butanoate metabolism, C5-Branched dibasic acid metabolism, Citrate cycle (TCA cycle), Alanine, aspartate and glutamate metabolism, Lipoic acid metabolism, Biosynthesis of kaempferol aglycones II, and Glutathione metabolism. These pathways were primarily related to flavonol glycoside metabolism, unsaturated fatty acid biosynthesis, organic acid metabolism, the TCA cycle, amino acid metabolism, and antioxidant metabolism (**Figure 2L**).

Across the six comparisons, pathways related to flavonoids and flavonol glycosides were repeatedly enriched, including Flavonoid biosynthesis, Isorhamnetin O-glycosides biosynthesis, Biosynthesis of flavonol glycosides, Biosynthesis of kaempferol aglycones II, Biosynthesis of quercetin aglycones I, and Flavone and flavonol biosynthesis. These pathways were particularly concentrated in TW vs. HN, YN vs. HN, and YN vs. TG, indicating that the differential metabolites in these comparisons were closely associated with flavonoid secondary metabolism. By contrast, the principal enriched pathways in JX vs. HN were Phenylpropanoid biosynthesis, Glucosinolate biosynthesis, and Phenylalanine metabolism, showing that the differential metabolites in this comparison were more broadly distributed across pathways associated with phenylpropanoid metabolism, glucosinolate metabolism, and aromatic amino acid metabolism.

Fatty acid metabolism-related pathways were also observed in multiple comparisons, including Linoleic acid metabolism, alpha-Linolenic acid metabolism, and Biosynthesis of unsaturated fatty acids. In TG vs. HN, Linoleic acid metabolism and alpha-Linolenic acid metabolism ranked among the major enriched pathways. Both alpha-Linolenic acid metabolism and Linoleic acid metabolism were identified in YN vs. HN, while Biosynthesis of unsaturated fatty acids ranked among the top ten pathways in YN vs. TG. These findings indicate that lipid metabolism-related pathways contributed to the differences between TG or YN and HN or TG.

Amino acid metabolism-related pathways were broadly distributed across the six comparisons, including Phenylalanine metabolism, Tyrosine metabolism, Valine, leucine and isoleucine biosynthesis, Valine, leucine and isoleucine degradation, Arginine biosynthesis, Arginine and proline metabolism, D-Amino acid metabolism, Alanine, aspartate and glutamate metabolism, 2-Oxocarboxylic acid metabolism, and Biosynthesis of amino acids. JX vs. HN mainly involved aromatic amino acid and branched-chain amino acid metabolism. Arginine biosynthesis was enriched in TG vs. HN, YN vs. HN, and YN vs. TW. Pathways related to arginine, proline, and glutamate metabolism were also identified in YN vs. TW and YN vs. TG, indicating that amino acid metabolism represented an important category of enriched pathways for the differential metabolites among the geographical groups.

Pathways associated with central carbon and energy metabolism mainly appeared in TW vs. HN, YN vs. TW, and YN vs. TG, including Pyruvate metabolism, Glyoxylate and dicarboxylate metabolism, Citrate cycle (TCA cycle), 2-Oxocarboxylic acid metabolism, Butanoate metabolism, and C5-Branched dibasic acid metabolism. Pyruvate metabolism and Glyoxylate and dicarboxylate metabolism ranked among the leading pathways in YN vs. TW. In YN vs. TG, Citrate cycle (TCA cycle), Butanoate metabolism, and C5-Branched dibasic acid metabolism were among the top ten enriched pathways, indicating that the differential metabolites in these comparisons were associated with carbon metabolism, organic acid metabolism, and energy metabolism.

Antioxidant and redox-related pathway enrichment was mainly reflected by the repeated occurrence of Glutathione metabolism. This pathway was included among the top ten enriched pathways in TG vs. HN, YN vs. HN, YN vs. TW, and YN vs. TG, indicating that differential metabolites associated with glutathione metabolism were enriched in multiple comparisons. In addition, cofactor-related pathways, including Pantothenate and CoA biosynthesis, Biosynthesis of cofactors, and Lipoic acid metabolism, were identified in several comparisons, mainly TG vs. HN, JX vs. HN, and YN vs. TG. These results suggest that cofactor biosynthesis and the associated enzymatic metabolic processes also contributed to the metabolic differences among some geographical groups.

Taken together, flavonoids represented one of the most prominent classes of geographically differential metabolites, and flavonoid- and flavonol-related pathways were repeatedly enriched across multiple pairwise comparisons. In particular, quercetin-related biosynthetic pathways were prominently represented, while quercetin- and naringenin-related glycosides, including Quercetin-3,7-Di-O-glucoside and Naringenin-4′-O-glucoside, were annotated among the differential metabolites. On the basis of these metabolomic observations, quercetin and naringenin were prioritized as representative flavonoid scaffolds for the subsequent exploratory network-pharmacology analysis. Their selection was not intended to indicate that they were the most abundant metabolites or the only potentially bioactive constituents of *A. pinnata* pith, but rather to provide representative flavonoid structures for hypothesis-generating investigation of possible human OA-related molecular targets.

### 3.2 Transcriptomic analysis

#### 3.2.1 Overall transcriptomic characteristics

In terms of the number of expressed genes, all populations exhibited relatively high levels of gene expression activity. The YN population had the largest number of detected expressed genes, whereas the TW population had comparatively fewer; however, the overall differences among populations were limited. This finding indicates that *A. pinnata* pith from different geographical origins is highly conserved in the maintenance of basic biological functions and shares a large number of genes involved in primary metabolism and development. Nevertheless, this overall consistency does not imply an absence of interpopulation differences. On the contrary, subsequent clustering and differential expression analyses revealed clear geographical differentiation in gene expression patterns.

Principal component analysis (PCA) provided an intuitive overview of the overall expression differences among the populations (**Figure 3A**). The first principal component (PC1) explained 34.99% of the total variance, while the second principal component (PC2) explained 16.78%, indicating that the PCA adequately captured the major expression differences among the populations. In the PCA score plot, the three biological replicates from each population clustered closely together, with no obvious outliers, demonstrating good experimental reproducibility and high data reliability. More importantly, populations from different geographical origins formed clearly separated clusters in the two-dimensional space. The HN and JX samples were both located in the negative region of PC1 and were positioned close to one another, indicating a high degree of similarity in gene expression between the Hainan and Jiangxi samples. The TW samples were clearly separated from HN and JX along PC2 and formed a relatively independent cluster. All TG samples were concentrated in the positive region of PC1 and were positioned far from the other populations, indicating the most pronounced transcriptomic differentiation. The YN samples were located between the TW and TG groups and exhibited an intermediate pattern. Collectively, this clustering pattern indicates that geographical origin has substantially influenced the gene expression profiles of *A. pinnata* pith. Among the five populations, the Thailand samples exhibited the most distinctive transcriptomic characteristics, whereas the Hainan and Jiangxi samples showed a relatively high degree of similarity.

**Figure 3:**
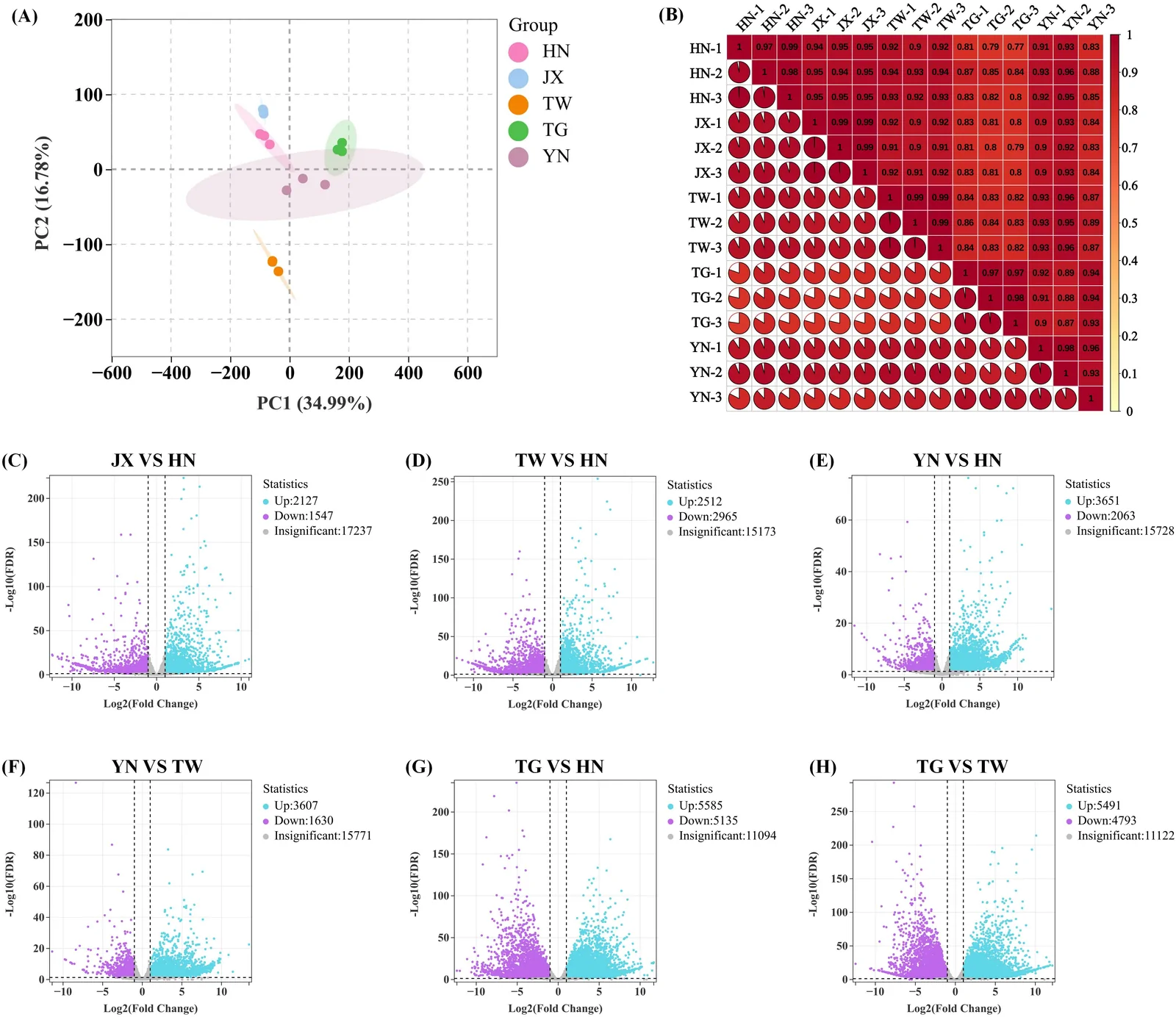
Transcriptomic analysis of *A. pinnata* pith from different geographical origins. [**(A)** Principal component analysis (PCA) of the transcriptomes of *A. pinnata* pith from different geographical origins; **(B)** correlation analysis among samples from different geographical origins; **(C)** volcano plot of differentially expressed genes in JX vs. HN; **(D)** volcano plot of differentially expressed genes in TW vs. HN; **(E)** volcano plot of differentially expressed genes in YN vs. HN; **(F)** volcano plot of differentially expressed genes in YN vs. TW; **(G)** volcano plot of differentially expressed genes in TG vs. HN; and **(H)** volcano plot of differentially expressed genes in TG vs. TW].

The sample correlation heatmap further supported the PCA results (**Figure 3B**). Biological replicates within each geographical group showed high pairwise correlations, indicating good within-group consistency. Based on the correlation coefficients displayed in the heatmap, the mean within-group correlations were approximately 0.980 for HN, 0.990 for JX, 0.990 for TW, 0.973 for TG, and 0.957 for YN. Although the YN group showed slightly greater within-group variation than the other groups, correlations among its biological replicates remained high.

At the interpopulation level, HN and JX showed the highest overall similarity, with a mean correlation coefficient of approximately 0.948 across their biological replicates. HN and TW also exhibited a relatively high mean correlation of approximately 0.926. In contrast, TG showed lower correlations with HN and JX, with mean coefficients of approximately 0.820 and 0.809, respectively, indicating greater transcriptomic differentiation of the Thailand population from these Chinese-origin populations. YN showed relatively high correlations with both TW and TG, with mean coefficients of approximately 0.921 and 0.909, respectively. This pattern was consistent with the intermediate position of the YN samples in the PCA plot.

In addition to the overall expression patterns, the composition of transcription factors also reflected potential differences in gene regulation among the populations. A total of 1,276 transcription factor genes were identified in this study, mainly belonging to the classical plant transcription factor families AP2/ERF, MYB, MYB-related, bHLH, NAC, WRKY, bZIP, C2H2, GRAS, and MADS-box. Among these, the AP2/ERF, MYB, and bHLH families contained the largest numbers of genes. These families are commonly involved in major biological processes, including plant growth and development, hormone signal transduction, stress responses, and the regulation of secondary metabolism. Because these transcription factors function upstream in regulatory networks, changes in their expression can induce extensive alterations in the expression of downstream genes. Therefore, the large-scale transcriptomic differences observed among the geographical populations were likely associated with the regulatory reprogramming of these key transcription factors. Overall, although the populations shared a broadly similar transcription factor composition, marked differences were already evident in the expression levels of individual family members, providing an important basis for subsequent differential expression analysis.

#### 3.2.2 Differential expression analysis

Differential expression analysis revealed substantial transcriptomic differentiation among *A. pinnata* pith samples from different geographical origins, and the extent of this differentiation was highly consistent with the sample clustering results. Using the Hainan population as the reference, only 3,674 differentially expressed genes (DEGs) were identified in JX vs. HN, representing the smallest difference among all comparisons (**Figure 3C**). Among these DEGs, 2,127 were upregulated and 1,547 were downregulated. Relative to the total number of expressed genes, this difference was limited, indicating that the Hainan and Jiangxi populations retained highly similar overall expression patterns. Combined with their close clustering in the PCA plot and their high correlation in the heatmap, these findings suggest that although *A. pinnata* populations from different regions of China are exposed to distinct environmental conditions, they retain relatively similar genetic backgrounds and regulatory patterns. Their transcriptomic differences may therefore be concentrated in a limited number of genes associated with environmental adaptation rather than reflecting genome-wide transcriptional reprogramming (The complete DEG datasets, together with the corresponding statistical information, are available through the public data link provided in the Data Availability statement).

By comparison, 5,477 DEGs were identified in TW vs. HN, including 2,512 upregulated and 2,965 downregulated genes, indicating a substantially greater degree of differentiation than that observed in JX vs. HN. These results suggest that the Taiwan population has developed a relatively distinct expression profile. Long-term geographical isolation from mainland China, together with the unique island climate of Taiwan, may have contributed to adaptive changes in specific regulatory networks and metabolic pathways, consistent with the independent distribution of the TW samples along PC2 in the PCA plot. Although TW and HN still showed a relatively high overall correlation, the large number of DEGs indicated considerable divergence in their gene expression profiles (**Figure 3D**).

A total of 5,714 DEGs were identified in YN vs. HN, including 3,651 upregulated and 2,063 downregulated genes. In addition, 5,237 DEGs were detected in YN vs. TW (**Figure 3F**), and 5,344 DEGs were identified in YN vs. TG. All three comparisons contained more than 5,000 DEGs, indicating a moderate degree of transcriptional differentiation between the Indonesian population and the other populations (**Figure 3E**). Notably, YN showed a relatively large number of upregulated genes in multiple comparisons, suggesting potentially greater metabolic activity or environmental responsiveness. Furthermore, the relatively high correlations of YN with both TW and TG support its intermediate position among the geographical populations, with the Indonesian population sharing certain transcriptomic characteristics with both the Chinese-origin and Southeast Asian-origin populations.

The most pronounced differences were observed in the TG-related comparisons. A total of 10,720 DEGs were identified in TG vs. HN, including 5,585 upregulated and 5,135 downregulated genes (**Figure 3G**). Similarly, 10,284 DEGs were detected in TG vs. TW, including 5,491 upregulated and 4,793 downregulated genes (**Figure 3H**). In both comparisons, the number of DEGs exceeded 10,000 and was substantially higher than that observed in the other comparisons, indicating extensive transcriptomic differentiation between the Thailand population and the Hainan and Taiwan populations. In the JX vs. HN volcano plot, most genes were concentrated around a log_2_FC value of zero, with relatively few genes located in the significantly upregulated or downregulated regions. In contrast, the TG vs. HN and TG vs. TW volcano plots showed extensive dispersion of genes toward both sides, reflecting large numbers of both upregulated and downregulated genes. This broad differential expression pattern suggests that the Thailand population did not simply enhance or suppress a specific functional gene group but instead underwent extensive remodeling of the overall regulatory network, involving numerous genes related to metabolism, signal transduction, and environmental responses.

Further analysis of shared and population-specific DEGs identified 701 genes that were differentially expressed across multiple comparisons using HN as the reference. These genes may represent core regulatory factors that commonly differ between the Hainan population and populations from other geographical origins. Notably, TG vs. HN contained 4,339 specific DEGs, substantially more than TW vs. HN (1,220), JX vs. HN (878), and YN vs. HN (537). The large number of specific DEGs in the Thailand population indicates that it has developed a distinctive gene-expression regulatory system and exhibits a greater degree of transcriptomic divergence than the other populations.

#### 3.2.3 GO functional classification

To further characterize the biological functions represented by differentially expressed genes in *A. pinnata* pith from different geographical origins, all expressed genes were subjected to Gene Ontology (GO) classification and KEGG pathway annotation. The results showed that these genes were widely involved in cellular organization, metabolism, environmental responses, signal transduction, and other biological processes, indicating that the expression differences among geographical populations were mainly associated with metabolic regulation and environmental adaptation. GO classification comprised three major categories: Biological Process, Cellular Component, and Molecular Function. Overall, the annotated genes displayed a clear hierarchical distribution, with terms related to metabolism and cellular activity predominating (**Figure 4**).

**Figure 4:**
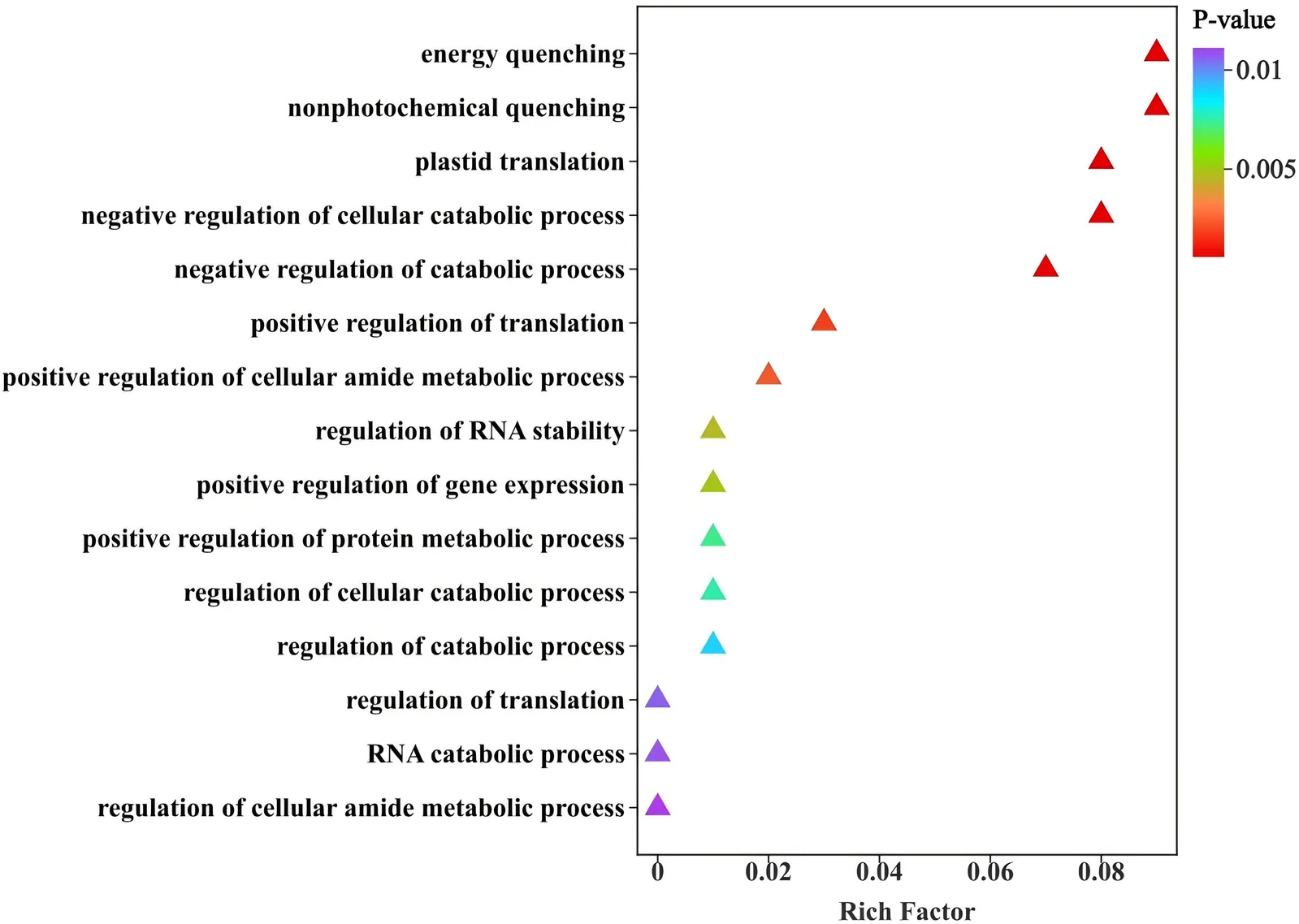
GO Analysis of *A. pinnata* Pith.

Within the Biological Process category, cellular process contained the largest number of genes, with 16,987 genes annotated, followed by metabolic process with 14,578 genes. These results indicate that most genes expressed in *A. pinnata* pith participate in normal cellular growth, development, and metabolism, which form the basis of plant survival and physiological activity. In addition, 7,547 genes were assigned to response to stimulus, indicating that numerous genes are involved in sensing and responding to environmental changes. Because this study compared *A. pinnata* pith samples from different geographical origins, these environment-responsive genes may represent an important basis for transcriptomic differentiation among populations. Notably, 4,181 genes were annotated to developmental process and 3,684 genes to multicellular organismal process, suggesting potential differences among populations in organ formation, growth, development, and morphogenesis. Such differences may be associated with long-term ecological adaptation and artificial selection in different regions.

Within the Cellular Component category, cellular anatomical entity contained 22,028 genes, representing the largest GO term. This finding indicates that most gene products are involved in the formation and maintenance of organelles, cell membranes, and intracellular structures. In addition, 3,798 genes were annotated to protein-containing complex, suggesting that numerous proteins function as components of complexes involved in cellular metabolism and signal transduction. These results indicate that although gene expression differed among geographical populations, their fundamental cellular structures and basic biological framework remained highly conserved.

Within the Molecular Function category, binding contained the largest number of genes, with 15,157 annotations, followed by catalytic activity with 11,458 genes. Binding functions mainly included DNA binding, RNA binding, protein binding, and ion binding, whereas catalytic activity was primarily associated with various enzymes. These findings indicate that a substantial proportion of genes in the *A. pinnata* pith transcriptome participate in metabolic reactions and the establishment of regulatory networks, thereby supporting normal physiological functions.

In addition, 1,958 genes were annotated to transcription regulator activity and 1,617 genes to transporter activity. These findings suggest that transcriptional regulation and substance transport are important mechanisms underlying the adaptation of *A. pinnata* pith to different ecological environments. Given the large number of differentially expressed genes observed among geographical populations, transcriptional regulators and transport proteins may constitute key functional modules driving environmental adaptation. Overall, the GO analysis indicated that the major functional differences among *A. pinnata* pith samples from different origins were concentrated in metabolic processes, environmental responses, biological regulation, and signal transduction, all of which are fundamental to plant adaptation to diverse ecological and climatic conditions.

### 3.3 PPI network analysis

Based on the STRING database, a protein-protein interaction network was constructed for candidate osteoarthritis-related targets regulated by quercetin. Of the 21 candidate proteins, 20 formed a highly connected subnetwork containing 55 interaction edges. The network density was 0.289, the average clustering coefficient was 0.567, and the average shortest path length was 2.0. Only F2 showed no direct interaction with other nodes at the selected threshold and therefore appeared as an isolated node (**Figure 5**).

**Figure 5:**
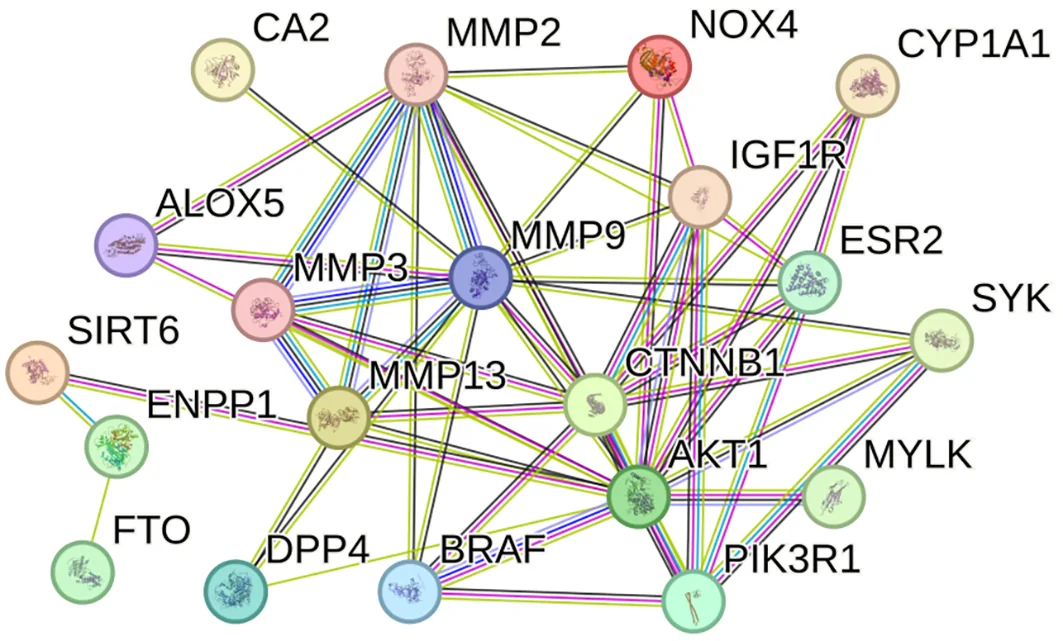
Protein-protein interaction network of osteoarthritis-related targets regulated by quercetin.

The overall network exhibited a pronounced modular organization and hub-driven topology. Based on interaction patterns and functional clustering, the network could be divided into three major functional modules, an extracellular matrix (ECM)/inflammatory remodeling module, a PI3K-AKT/Wnt signaling module, and a metabolic/regulatory module. The ECM/inflammatory remodeling module included MMP2, MMP3, MMP9, MMP13, NOX4, and ALOX5 and displayed the highest level of internal connectivity. The PI3K-AKT/Wnt signaling module was centered on CTNNB1 and PIK3R1, which formed a signaling network with IGF1R, ESR2, BRAF, SYK, and other nodes. The metabolic/regulatory module comprised SIRT6, ENPP1, and FTO. Although relatively independent, this module remained connected to the signaling module through a limited number of interactions.

Network centrality analysis identified AKT1 (degree = 16), MMP9 (degree = 13), CTNNB1 (degree = 11), and MMP2 (degree = 10) as the principal hub nodes. These proteins occupied central positions within the network and served as connectors among different functional modules. Their high degree of connectivity suggests that they may play important roles in integrating information across the network. In contrast, F2 did not form any effective interactions at the selected threshold and remained an isolated node.

A protein-protein interaction (PPI) network of candidate osteoarthritis-related targets regulated by naringenin was constructed using the STRING database. Of the 26 candidate proteins, 25 formed a highly interconnected PPI network containing 127 protein-protein interactions, with a network density of 0.423, indicating strong connectivity among the candidate targets. Topological analysis revealed a pronounced hub-centered structure. MMP9 and AKT1 exhibited the highest degree values (Degree = 20) and were identified as the principal hub nodes, followed by PPARG (Degree = 18) and PTGS2 (Degree = 17), which also showed high network centrality. In addition, MMP2, BCL2, ESR1, and SERPINE1 displayed relatively high connectivity and contributed to important regulatory modules within the network (**Figure 6**).

**Figure 6:**
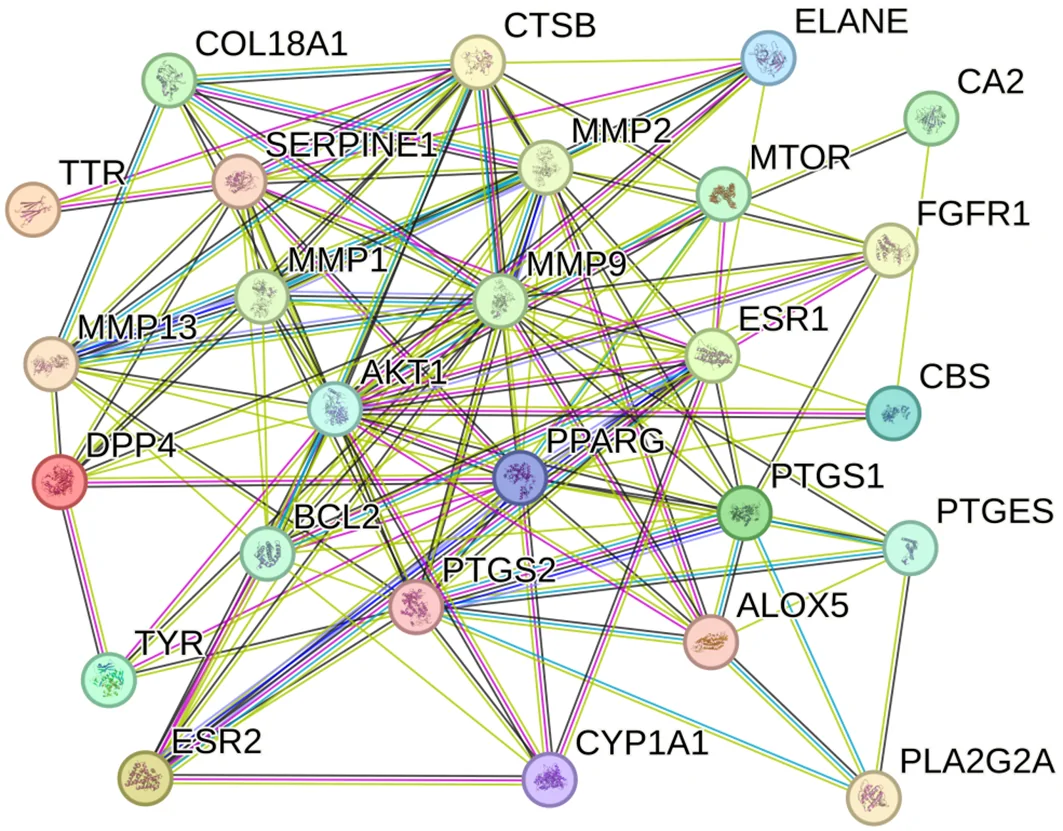
Protein-protein interaction network of osteoarthritis-related targets regulated by naringenin.

Functional module analysis showed that the network was mainly associated with extracellular matrix remodeling, inflammatory regulation, and metabolic signaling. Matrix metalloproteinases, including MMP1, MMP2, MMP9, and MMP13, formed a closely connected extracellular matrix degradation module. PTGS1, PTGS2, ALOX5, PLA2G2A, and PTGES constituted an inflammation- and lipid metabolism-related module, whereas AKT1, MTOR, PPARG, and FGFR1 formed a signaling and metabolic regulatory module. Overall, the network analysis indicated that naringenin may regulate osteoarthritis through multiple interconnected targets, among which MMP9, AKT1, PTGS2, PPARG, MMP2, and MMP13 may serve as key regulatory targets.

### 3.4 Machine learning analysis

Differential expression analysis (**Figure 7A**) identified 2,011 differentially expressed genes (DEGs) using the criteria of FDR < 0.05 and |log_2_FC| ≥ 1. Among these, 1,079 genes were significantly upregulated in the OA group, accounting for 53.65%, whereas 932 genes were significantly downregulated, accounting for 46.35%. These results indicate extensive and significant transcriptomic remodeling in OA cartilage. The volcano plot further showed that many DEGs exhibited both large expression fold changes and high statistical significance. CFI was located in the significantly upregulated region, with a log_2_FC of 3.340 and an FDR of 9.38 × 10^-32^. By contrast, DDIT3, MAFF, and KLF10 were significantly downregulated, with log_2_FC values of -2.782, -3.124, and -2.310 and corresponding FDR values of 5.40 × 10^-70^, 2.85 × 10^-52^, and 1.16 × 10^-36^, respectively. These values were all far below 0.05, indicating both substantial expression changes and extremely high statistical reliability.

**Figure 7:**
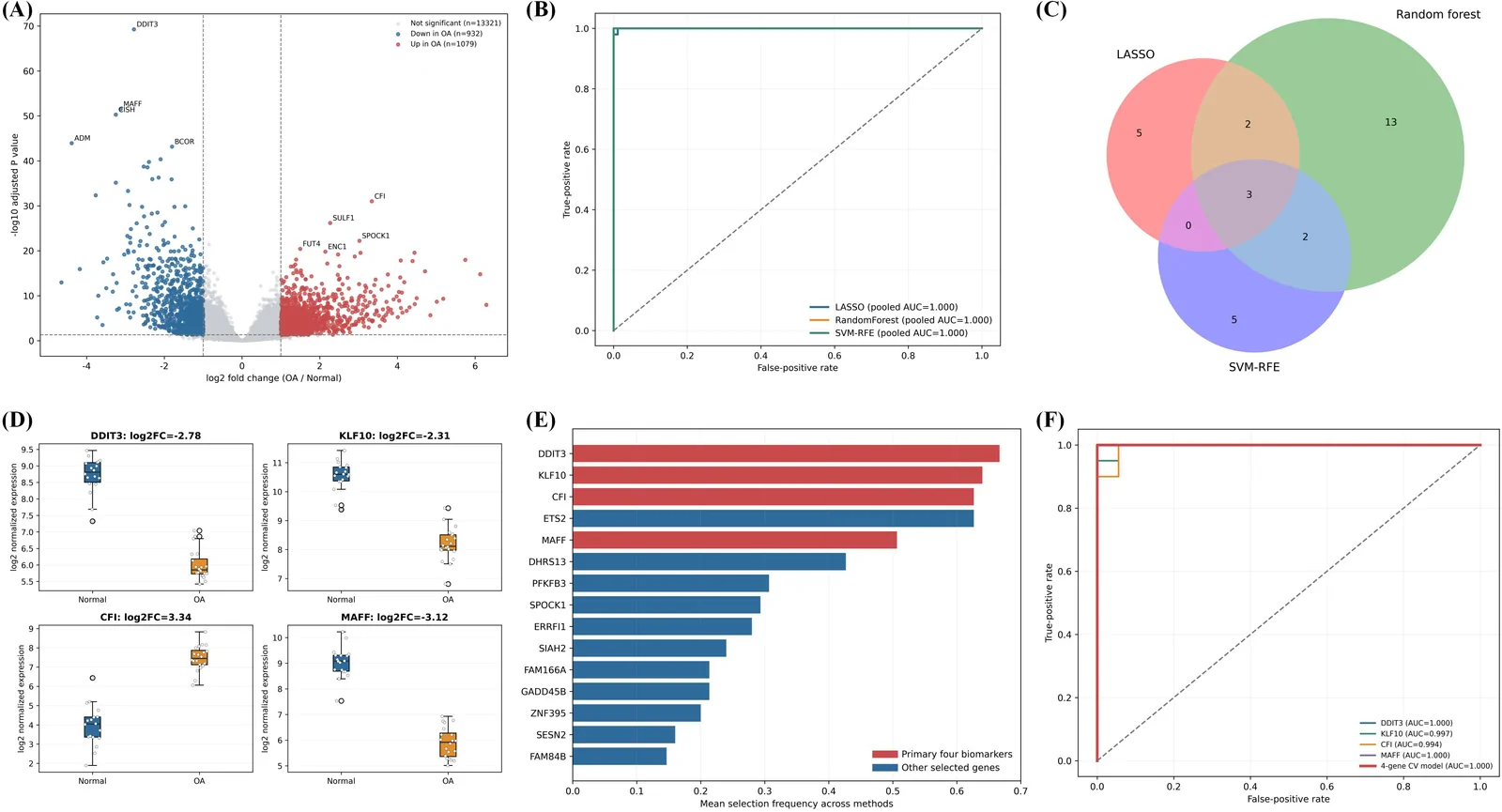
Machine learning analysis of osteoarthritis GEO datasets. [**(A)** Differential expression analysis; **(B)** Cross-validation results; **(C)** Overlap of candidate genes identified by different machine-learning algorithms; **(D)** Expression distribution of candidate biomarkers; **(E)** Feature selection stability; **(F)** ROC analysis of individual biomarkers and the combined diagnostic model].

The cross-validation results (**Figure 7B**) further demonstrated the excellent classification performance of the machine-learning models. The receiver operating characteristic (ROC) curves of the LASSO, Random Forest, and SVM-RFE models were all positioned close to the upper-left corner, and the summarized areas under the ROC curve (AUCs) reached 1.000 for all three models. Comparison of the evaluation metrics showed that Random Forest achieved the best overall performance, with an ROC AUC, balanced accuracy, sensitivity, specificity, and F1 score of 1.000. LASSO and SVM-RFE also achieved ROC AUC values of 1.000, balanced accuracy values of 0.993, sensitivities of 1.000, specificities of 0.987, and F1 scores of 0.996. The repeated cross-validation analysis yielded high apparent discriminatory performance within the GSE114007 cohort. The mean ROC AUC values were close to 1.0 for the three machine-learning approaches. However, given the limited sample size and the absence of an independent external validation cohort, these estimates should be interpreted as internal performance rather than evidence of established diagnostic accuracy.

Intersection analysis of the genes selected by the three machine-learning algorithms (**Figure 7C**) showed that LASSO, Random Forest, and SVM-RFE identified 10, 20, and 10 candidate feature genes, respectively. Venn analysis revealed that three genes, DDIT3, KLF10, and CFI, were selected by all three algorithms, accounting for 42.86% of the overlapping candidate genes. An additional four genes, including MAFF, were identified by at least two algorithms, resulting in a final set of seven overlapping candidate genes. Although each algorithm independently identified several algorithm-specific genes, only a limited number were repeatedly selected across different methods. This suggests that the overlapping genes showed relatively high selection stability across the applied machine-learning approaches and were therefore retained as candidate OA-associated biomarkers for further investigation.

Further analysis of feature-selection stability across repeated cross-validation runs (**Figure 7E**) showed that DDIT3, KLF10, and CFI were consistently selected by all three machine-learning algorithms, with mean selection-stability scores of 0.667, 0.640, and 0.627, respectively, the highest among all candidate genes. MAFF was selected by two algorithms and had a mean stability score of 0.507. All four genes also exhibited substantial expression changes. CFI was significantly upregulated in the OA group, with a log_2_FC of 3.340, whereas DDIT3, KLF10, and MAFF were significantly downregulated, with log_2_FC values of -2.782, -2.310, and -3.124, respectively. In terms of single-gene diagnostic performance, DDIT3 and MAFF each achieved an ROC AUC of 1.000, while KLF10 and CFI achieved AUC values of 0.997 and 0.994, respectively. These results indicate that the four genes showed consistent feature-selection patterns and high discriminatory performance within the GSE114007 dataset and were therefore prioritized as candidate OA-associated biomarkers for further validation.

**Figure 7D** further illustrates the expression distributions of the four candidate biomarkers in the normal and OA groups. DDIT3, KLF10, and MAFF were all expressed at substantially lower levels in the OA group than in the normal group, with expression decreases of approximately 6.9-fold, 4.96-fold, and 8.0-fold, respectively. In contrast, CFI expression was markedly increased by approximately 10.1-fold in the OA group. The boxplots showed clear separation between the normal and OA groups for all four genes, with minimal overlap and only a few outliers, indicating stable and consistent expression patterns that effectively reflect the molecular alterations associated with OA cartilage.

The diagnostic performance of the four biomarkers was further evaluated using single-gene and combined-model ROC analyses (**Figure 7F**). Single-gene ROC analysis showed that DDIT3 and MAFF each achieved an AUC of 1.000, indicating perfect d iscrimination between OA and normal samples in the current dataset. KLF10 and CFI also exhibited excellent diagnostic performance, with AUC values of 0.997 and 0.994, respectively. Based on these four biomarkers, a combined diagnostic model was established, which also achieved an ROC AUC of 1.000 during repeated cross-validation. Its ROC curve closely approached the upper-left corner, indicating 100% sensitivity and specificity and excellent internal discriminatory performance. Collectively, the differential-expression, feature-selection, and ROC analyses identified DDIT3, KLF10, CFI, and MAFF as candidate OA-associated biomarkers in the GSE114007 dataset. Their diagnostic relevance requires confirmation in independent datasets and clinical cohorts.

### 3.5 Molecular docking

Molecular docking predicted that quercetin and naringenin could adopt favorable binding poses within the selected binding regions of AKT1 and MMP9. The predicted optimal docking scores were −8.6 kcal/mol for the quercetin–AKT1 complex, −7.8 kcal/mol for the naringenin–AKT1 complex, −9.1 kcal/mol for the quercetin–MMP9 complex, and −8.2 kcal/mol for the naringenin–MMP9 complex. Under the present docking conditions, quercetin yielded more favorable docking scores than naringenin for both AKT1 and MMP9. These values represent computational docking scores rather than experimentally measured binding affinities.

In the AKT1 complex, quercetin was predicted to interact primarily with the amino acid residues Trp80, Glu85, Ser205, Leu210, Thr211, Lys268, Tyr272, Arg273, and Asp274. The multiple hydroxyl groups and planar aromatic scaffold of quercetin facilitate the formation of an extensive hydrogen-bonding network and π-related interactions within the allosteric binding pocket of AKT1, consistent with its favorable predicted docking score (-8.6 kcal/mol) (**Figures 8A–C**).

**Figure 8:**
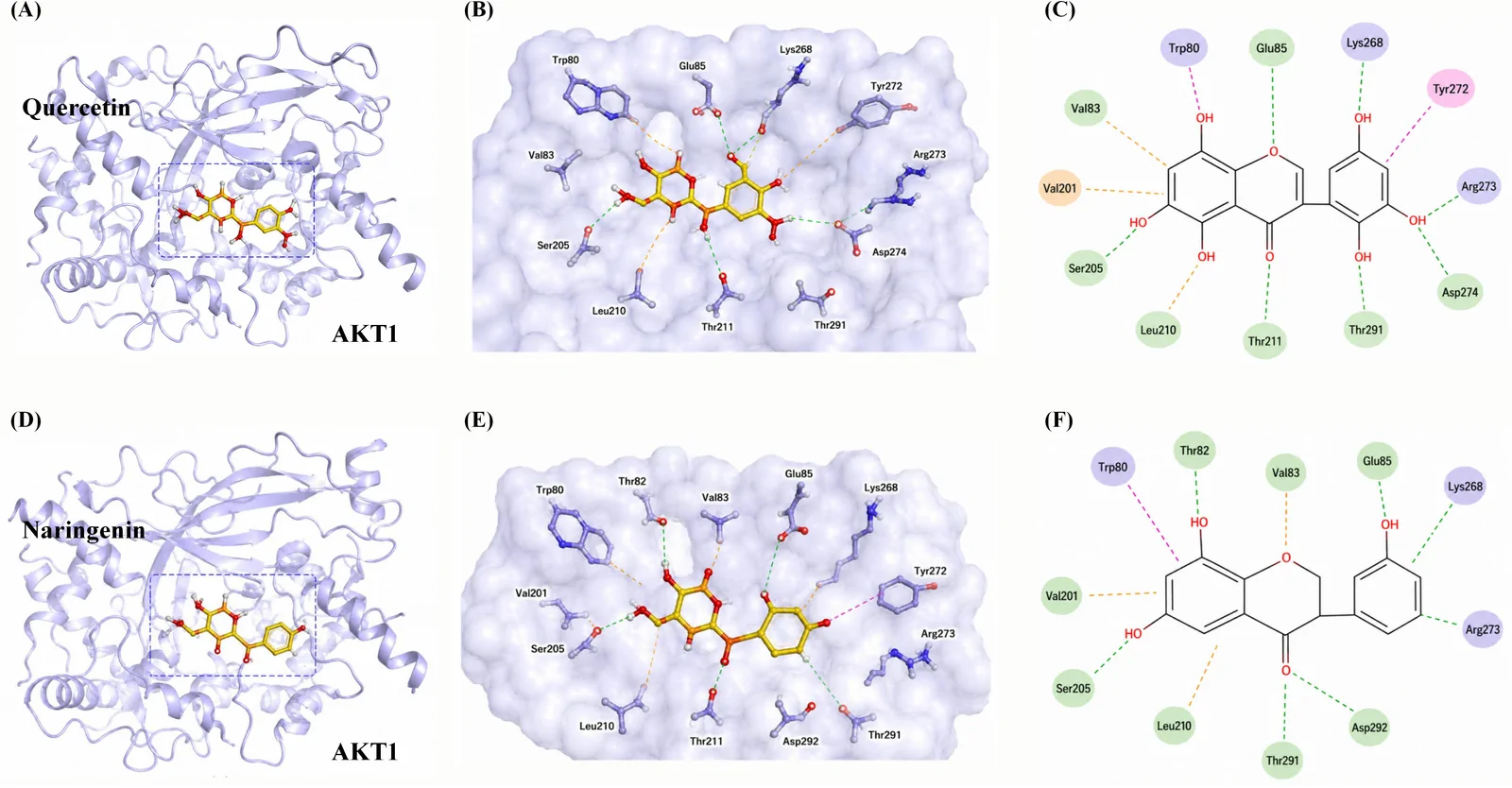
Binding of quercetin and naringenin to AKT1.

In contrast, naringenin was predicted to bind to a partially overlapping binding region involving Trp80, Thr82, Val83, Glu85, Ser205, Leu210, Thr211, Lys268, Tyr272, Arg273, Thr291, and Asp292. As a flavanone, naringenin possesses a more flexible molecular scaffold than quercetin, which may enable it to adapt more effectively to adjacent sub-binding pockets through conformational adjustment and thereby establish stable protein-ligand interactions. Nevertheless, its predicted binding affinity (-7.8 kcal/mol) was slightly lower than that of quercetin (**Figures 8D–F**).

For MMP9, both ligands were predicted to bind near the catalytic pocket containing the Zn^2+^ ion. Quercetin was predicted to interact with key amino acid residues, including His401, Glu402, His405, His411, Gln192, Leu188, Ala189, Pro421, Tyr423, and Arg424. Owing to its abundance of oxygen-containing functional groups, quercetin may approach the catalytic Zn²^+^ ion and further stabilize protein-ligand binding through hydrogen bonding and hydrophobic interactions. Consequently, it achieved the most favorable predicted docking score among the four complexes (-9.1 kcal/mol) (**Figures 9A–C**).

**Figure 9:**
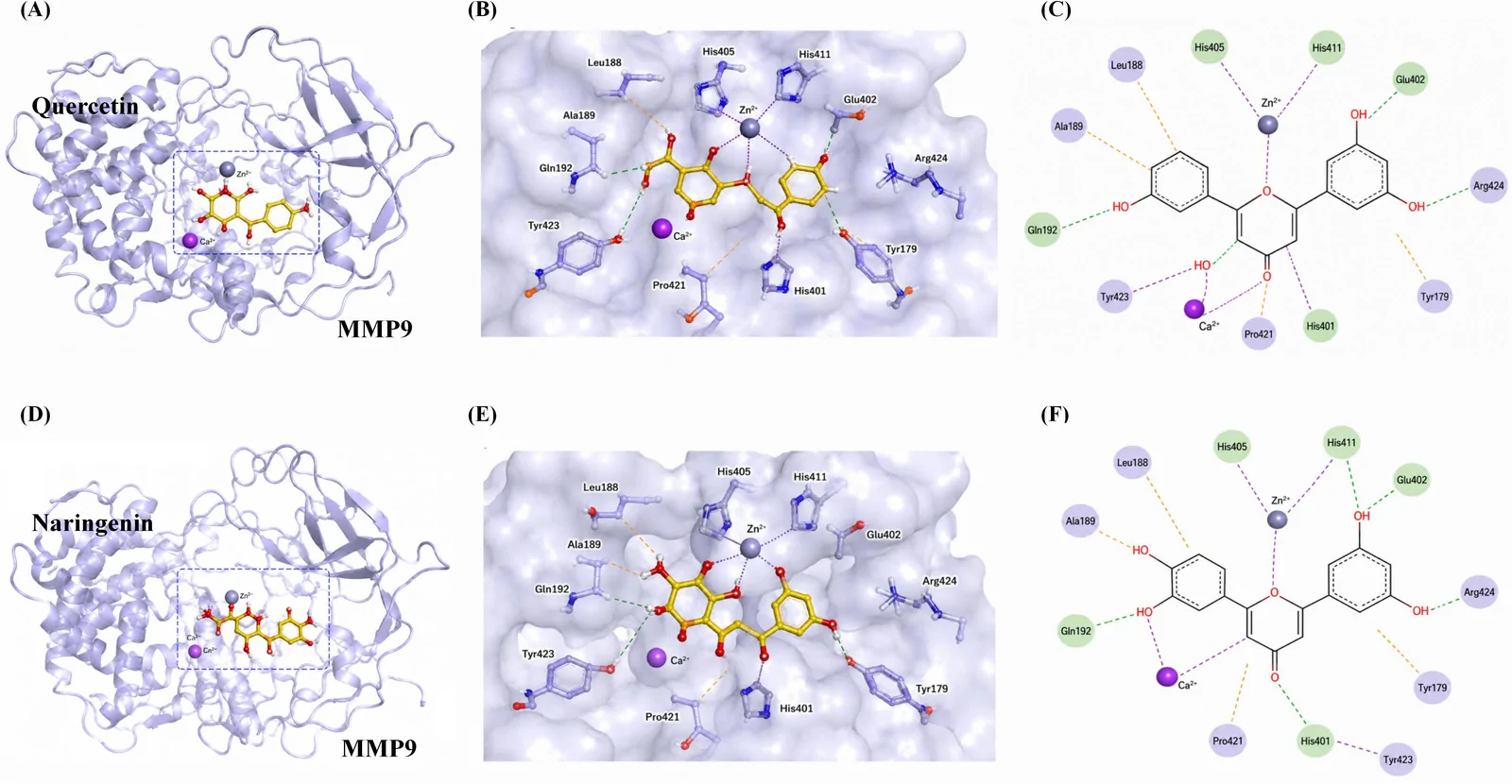
Binding of quercetin and naringenin to MMP9.

Naringenin was likewise predicted to bind near the catalytic center of MMP9 and formed a partially overlapping interaction network with many of the same residues. However, compared with quercetin, its binding mode appeared to rely more on conformational adaptability and occupation of hydrophobic sub-pockets, resulting in a slightly lower predicted docking score of -8.2 kcal/mol (**Figures 9D–F**).

Overall, quercetin, with its greater number of hydroxyl groups and higher molecular planarity, may exhibit stronger polar interactions and greater potential for metal ion-associated interactions. In contrast, the higher molecular flexibility of naringenin may facilitate adaptation to different binding conformations.

## 4 Discussion

### 4.1 Geographical origin is associated with differences in metabolite accumulation and gene expression patterns in *A. pinnata* pith

Distinct geographical origins resulted in substantial differences in the metabolite composition, relative abundance, and gene expression profiles of *A. pinnata* pith. Overall metabolomic analysis showed that samples from Taiwan, China contained the greatest diversity of detected metabolites and exhibited pronounced accumulation of flavonoids, phenolic acids, and amino acid derivatives. Although the Jiangxi samples contained relatively fewer detected metabolites overall, they showed elevated levels of specific flavonoids and lipids. In contrast, the Hainan samples displayed a relative advantage in the accumulation of amino acids, organic acids, free fatty acids, and certain lignans. Notably, a total of 208 differential flavonoids were identified between the Taiwan and Hainan samples, of which 178 were upregulated in the Taiwan samples. Phenolic acids and amino acid derivatives also exhibited an overall upregulation trend (Meijon et al., 2016).

These geographical differences may reflect the combined influence of genetic background and environmental conditions. However, because these factors were not experimentally controlled or independently quantified in the present study, their individual contributions to the observed metabolomic and transcriptomic variation cannot be determined. Environmental factors such as temperature, precipitation, light intensity, ultraviolet radiation, soil moisture, mineral nutrition, and salinity can alter carbon and nitrogen allocation, redox homeostasis, and defense responses in plants, thereby regulating the biosynthesis and accumulation of secondary metabolites, including phenolics, flavonoids, terpenoids, and alkaloids (Upadhyay et al., 2025). The effects of these environmental factors are rarely linear and are often species-, developmental stage-, and tissue-specific. Consequently, the same class of metabolites may exhibit distinct or even opposite accumulation patterns across different geographical origins.

The transcriptomic results further confirmed the influence of geographical origin on the molecular phenotypes of *A. pinnata* pith. The overall gene expression profiles of the Hainan and Jiangxi samples were relatively similar, whereas 10,720 and 10,284 differentially expressed genes (DEGs) were identified between the Thailand samples and the Hainan and Taiwan samples, respectively, indicating extensive transcriptomic reprogramming in the Thailand population. Moreover, the Thailand samples contained a large number of origin-specific DEGs involved in metabolism, signal transduction, and environmental response. In contrast, the Indonesia samples exhibited an intermediate expression pattern, sharing transcriptomic characteristics with both the Chinese and Southeast Asian populations.

Notably, the degree of transcriptomic divergence was not fully consistent with the overall abundance of metabolites. For example, although the Thailand samples exhibited the largest number of DEGs, they did not show the highest overall metabolite accumulation. This observation suggests that metabolite abundance is regulated not only at the transcriptional level but also by multiple downstream processes, including enzyme activity, post-translational modification, substrate availability, metabolite transport, and degradation. Therefore, the transcriptomic and metabolomic differences observed in this study should be interpreted as biological responses occurring at different regulatory levels rather than assuming that a greater number of DEGs necessarily corresponds to higher levels of bioactive metabolites.

In addition, the present study was based on naturally collected materials from different geographical regions. Consequently, geographical origin may be confounded with factors such as genetic background, tree age, tissue developmental and physiological status, and cultivation practices. Therefore, while the current results demonstrate stable differences in metabolite accumulation and gene expression among populations from different origins, these differences cannot yet be attributed exclusively to any single environmental factor. Future studies incorporating common-garden experiments, cultivation of identical genotypes across multiple locations, and multi-year sampling will help disentangle the respective contributions of genetic effects, environmental effects, and genotype-by-environment interactions. However, the present study does not establish direct causal relationships between individual metabolite changes and gene-expression differences.

### 4.2 Flavonoid-phenylpropanoid metabolism and redox regulation constitute the core metabolic basis underlying geographical variation in *A. pinnata* pith

#### 4.2.1 Flavonoid and phenylpropanoid metabolism represent the major differential pathways among *A. pinnata* pith from different geographical origins

KEGG enrichment analysis of differential metabolites revealed that metabolites from *A. pinnata* pith collected from different geographical origins were consistently enriched in pathways related to the biosynthesis of plant secondary metabolites, phenylpropanoid biosynthesis, flavonoid biosynthesis, flavonol glycoside biosynthesis, isorhamnetin glycoside biosynthesis, and quercetin- and kaempferol-related metabolic pathways. These pathways were repeatedly identified in comparisons between Taiwan and Hainan, Indonesia and Hainan, and Indonesia and Thailand, suggesting that the flavonoid-phenylpropanoid metabolic axis represents the core metabolic module underlying the geographical variation in the chemical composition of *A. pinnata* pith (Liu et al., 2021).

Flavonoids are synthesized through the phenylpropanoid pathway. Following the deamination of phenylalanine by upstream enzymes such as phenylalanine ammonia-lyase, *p*-coumaroyl-CoA is produced and subsequently converted into diverse flavonoid subclasses, including flavanones, flavones, flavonols, and anthocyanins, through the coordinated actions of chalcone synthase, chalcone isomerase, various hydroxylases, reductases, and glycosyltransferases (Liu et al., 2021). The observed differential flavonoid accumulation is consistent with variation in phenylpropanoid- and flavonoid-related metabolism. However, because metabolic flux, enzyme activity, and substrate allocation were not directly measured, coordinated regulation of these processes cannot be inferred directly from the present data.

Among the geographical populations examined, the Taiwan samples exhibited the most pronounced flavonoid differences relative to the Hainan samples. Of the 208 differential flavonoids identified, 178 were upregulated, and phenolic acids also showed an overall increasing trend. These findings suggest that the Taiwan population may allocate a greater proportion of phenylpropanoid precursors toward the biosynthesis of flavonoids and related phenolic compounds. Compared with the Hainan and Thailand populations, the Indonesia samples also exhibited increased accumulation of flavonoids, flavonol glycosides, and phenolic acids. However, relative to the Taiwan samples, most flavonoids and amino acid derivatives were reduced. This pattern suggests that the Taiwan population possesses a stronger overall capacity for flavonoid accumulation, whereas the Indonesia population is characterized by preferential accumulation of specific flavonoid subclasses together with a more balanced metabolic profile.

The Jiangxi population displayed a distinct metabolic allocation pattern. Although most differential metabolites were downregulated relative to the Hainan population, 113 of the 169 differential flavonoids were upregulated. This finding suggests that, despite relatively lower overall metabolic activity, the Jiangxi population may preferentially maintain or enhance specific branches of flavonoid metabolism.

#### 4.2.2 Primary metabolism, lipid metabolism, and antioxidant metabolism cooperatively contribute to geographical adaptation

In addition to flavonoid and phenylpropanoid pathways, this study identified significant enrichment of amino acid metabolism, fatty acid metabolism, central carbon metabolism, and glutathione metabolism across multiple pairwise comparisons. Metabolic pathways involving phenylalanine, tyrosine, arginine, proline, glutamate, and branched-chain amino acids were enriched in several comparison groups. In contrast, pyruvate metabolism, the tricarboxylic acid (TCA) cycle, and glyoxylate and dicarboxylate metabolism were primarily enriched in the comparisons between Indonesia and Taiwan and between Indonesia and Thailand.

The concurrent enrichment of secondary-metabolism, amino-acid, and central-carbon metabolic pathways suggests that these processes may be associated across geographical groups. However, the present pathway-enrichment analysis does not establish direct metabolic coupling or causal interactions among these pathways. Amino acids serve not only as building blocks for protein synthesis but also as precursors for numerous alkaloids, phenolic compounds, and other nitrogen-containing secondary metabolites. Likewise, pyruvate metabolism and the TCA cycle provide carbon skeletons, reducing equivalents, and ATP required for secondary metabolite biosynthesis. Under different environmental conditions, plants may reallocate resources between growth-related metabolism and defense-related metabolism, thereby generating distinct metabolite profiles (Mandal et al., 2022; Zhang et al., 2026).

Lipid metabolism also represents an important component of geographical variation. Linoleic acid metabolism, α-linolenic acid metabolism, and unsaturated fatty acid biosynthesis were repeatedly enriched in comparisons involving the Thailand and Indonesia populations. Fatty acids are not only essential structural components of cellular membranes but also serve as signaling molecules and precursors for plant hormones such as jasmonates. Under different temperature and water availability conditions, plants can regulate membrane fluidity by altering the proportion of unsaturated fatty acids. Therefore, the observed differences in lipid metabolism may be associated with temperature adaptation and stress responses among *A. pinnata* populations from different geographical origins. However, because membrane properties and phytohormone levels were not directly measured in this study, this interpretation requires further experimental validation (Rehman et al., 2023).

Glutathione metabolism was repeatedly enriched in the comparisons between Thailand and Hainan, Indonesia and Hainan, Indonesia and Taiwan, and Indonesia and Thailand, suggesting that redox regulation represents a common biological process underlying geographical variation in *A. pinnata* pith. Glutathione is a key antioxidant responsible for maintaining cellular redox homeostasis and scavenging reactive oxygen species in plants, and alterations in its metabolism are closely associated with environmental stress responses, antioxidant defense, and the regulation of secondary metabolism (Zhang et al., 2026). The concurrent changes in glutathione metabolism and flavonoid accumulation further suggest that *A. pinnata* populations from different geographical origins may maintain redox homeostasis through coordinated regulation of the enzymatic antioxidant system and non-enzymatic antioxidant metabolites.

### 4.3 Predicted OA-related target networks of quercetin and naringenin involve inflammation, extracellular matrix remodeling, and cell-survival pathways

Given the prominent geographical variation in flavonoid metabolism and the occurrence of quercetin- and naringenin-related derivatives in the metabolomic dataset, quercetin and naringenin were selected as representative flavonoid scaffolds for exploratory evaluation of their predicted human OA-related target networks. These analyses were intended to generate mechanistic hypotheses rather than to establish the pharmacological activity of the compounds in *A. pinnata* pith.

#### 4.3.1 The PPI networks suggest that extracellular matrix degradation and inflammatory signaling are the major functional modules

Osteoarthritis (OA) is not merely a degenerative disease characterized by cartilage wear but a complex joint disorder involving chondrocyte metabolic imbalance, extracellular matrix (ECM) degradation, synovial inflammation, subchondral bone remodeling, and cell death (Huang et al., 2025). Pro-inflammatory cytokines induce the expression of matrix-degrading enzymes, including matrix metalloproteinases (MMPs) and ADAMTS family members, leading to the degradation of type II collagen and aggrecan. The resulting degradation products further stimulate inflammatory responses, thereby establishing a self-perpetuating cycle of inflammation and matrix destruction (De Roover et al., 2023).

The quercetin-associated protein-protein interaction (PPI) network constructed in this study showed that 20 of the 21 candidate proteins formed a highly interconnected network, with AKT1, MMP9, CTNNB1, and MMP2 serving as the principal hub nodes. This network could be broadly divided into functional modules associated with extracellular matrix remodeling and inflammation, PI3K-AKT/Wnt signaling, and metabolic regulation. The naringenin-associated network exhibited even greater connectivity, with 25 of 26 candidate proteins forming a network containing 127 protein-protein interactions. MMP9 and AKT1 displayed the highest degree values, while PPARG, PTGS2, MMP2, and MMP13 also occupied central positions within the network.

MMP2, MMP9, and MMP13 collectively represent a major extracellular matrix degradation module. Among these, MMP13 is widely recognized as a key enzyme responsible for the degradation of type II collagen in OA cartilage, whereas MMP9 participates not only in the degradation of gelatin and extracellular matrix proteins but also in the activation of inflammatory mediators and tissue remodeling (Wolosowicz et al., 2025). In the present study, MMP9 emerged as a hub node in both the quercetin- and naringenin-associated networks, suggesting that suppression of excessive extracellular matrix degradation may represent a shared OA-related molecular process highlighted by the predicted target networks of these two compounds.

Proteins including PTGS2, ALOX5, PLA2G2A, and PTGES were primarily associated with arachidonic acid metabolism and inflammatory lipid mediator biosynthesis. PTGS2 encodes cyclooxygenase-2 (COX-2), a key enzyme involved in prostaglandin production that is closely associated with synovial inflammation and pain in OA. The relatively high connectivity of PTGS2 in the naringenin-associated network suggests that the potential effects of naringenin may extend beyond cartilage protection to include regulation of inflammatory lipid mediators (Dvorshchenko et al., 2025).

AKT1 occupied a central position in both PPI networks, linking signaling modules involved in cell survival, autophagy, metabolism, and inflammation. However, the role of AKT signaling in OA is highly context-dependent. Depending on cell type, disease stage, and upstream or downstream signaling context, AKT activation may produce distinct biological effects. Therefore, the present findings only suggest that AKT1 is an important network-associated target. The PPI and molecular docking analyses alone are insufficient to determine whether quercetin or naringenin directly activates or inhibits AKT1 (Sun et al., 2020).

#### 4.3.2 Quercetin may exert anti-osteoarthritis effects through anti-inflammatory, antioxidant, and cartilage microenvironment-modulating mechanisms

Quercetin possesses multiple phenolic hydroxyl groups and a conjugated aromatic scaffold, endowing it with strong hydrogen-bonding capability and considerable redox-regulating potential. Previous animal and cellular studies have demonstrated that quercetin reduces the expression of IL-1β and TNF-α while suppressing TLR4/NF-κB signaling, thereby alleviating joint inflammation and tissue damage in OA models (Zhang et al., 2020). Other studies have further shown that quercetin attenuates oxidative stress, endoplasmic reticulum stress, and apoptosis in chondrocytes through SIRT1/AMPK-related mechanisms (Feng et al., 2019).

The biological effects of quercetin may also involve regulation of the interaction between chondrocytes and the synovial immune microenvironment. Hu et al. reported that quercetin suppressed IL-1β-induced expression of matrix-degrading enzymes and inflammatory mediators, reduced reactive oxygen species production and apoptosis in chondrocytes, and promoted the polarization of synovial macrophages toward an M2-like phenotype, thereby creating a local microenvironment favorable for cartilage synthesis and repair (Hu et al., 2019). The same study also demonstrated that quercetin modulated the Akt/NF-κB signaling pathway, which is consistent with the central position of AKT1 observed in the PPI network of the present study.

However, this consistency merely indicates that both experimental evidence and network analysis converge on inflammation- and cell survival-related signaling pathways. It does not demonstrate that quercetin present in *A. pinnata* necessarily exerts its biological effects through the same mechanisms. In particular, *A. pinnata* may contain quercetin aglycone, quercetin glycosides, and other coexisting phytochemicals. These different chemical forms may vary substantially in cellular uptake, metabolic stability, and target-binding affinity. Therefore, future studies should first accurately identify and quantify quercetin and its derivatives in *A. pinnata* and subsequently evaluate their anti-osteoarthritis activities individually.

#### 4.3.3 Potential OA-related molecular actions of quercetin suggested by previous studies and network analysis

The naringenin-associated PPI network identified in this study exhibited pronounced multi-target characteristics, encompassing extracellular matrix-degrading proteins such as MMP1, MMP2, MMP9, and MMP13; inflammation-related proteins including PTGS2, ALOX5, and PLA2G2A; as well as regulatory nodes involved in metabolism and cell fate, such as AKT1, MTOR, and PPARG. This network architecture suggests that the predicted OA-related targets associated with naringenin are involved in inflammatory responses, extracellular matrix homeostasis, and chondrocyte-survival pathways.

Previous experimental studies provide supporting evidence for this hypothesis. Wang et al. demonstrated that naringenin dose-dependently reduced IL-6, TNF-α, and COX-2 production in IL-1β-stimulated chondrocytes, suppressed MMP3, MMP9, MMP13, and ADAMTS5, reduced chondrocyte apoptosis, and preserved type II collagen. These effects were associated with inhibition of the TLR4/TRAF6/NF-κB pathway. In a mouse OA model, naringenin also attenuated inflammatory responses and extracellular matrix degradation (Wang et al., 2023).

These findings are consistent with the prominent positions of MMP9, PTGS2, and inflammation-related signaling nodes observed in the present PPI network, suggesting that the potential anti-OA activity of naringenin may involve at least three interconnected mechanisms. First, naringenin may suppress inflammatory signaling pathways, such as TLR4/NF-κB, thereby reducing the production of pro-inflammatory cytokines and inflammatory enzymes. Second, it may decrease the expression of MMPs and ADAMTS family members, thereby slowing the degradation of type II collagen and proteoglycans. Finally, by attenuating oxidative stress and chondrocyte apoptosis, naringenin may preserve chondrocyte viability and maintain cartilage anabolic function.

### 4.4 Study limitations and future perspectives

Several limitations of the present study should be acknowledged. First, the samples originated from different geographical regions and may differ simultaneously in genetic background, environmental conditions, developmental status, and cultivation practices. Therefore, although clear metabolomic and transcriptomic differences were observed among geographical groups, the present study cannot distinguish the relative contributions of genetic and environmental factors. Future studies should incorporate common-garden experiments, detailed environmental measurements, population-genetic analyses, and multi-year sampling to determine the stability and major drivers of the observed geographical variation.

Second, the connection between the plant metabolomic dataset and the subsequent OA-related computational analyses remains exploratory. The untargeted metabolomic dataset contained flavonoid-related compounds, including glycosylated and conjugated derivatives; however, the present analysis does not establish the presence or concentration of free quercetin and naringenin aglycones at pharmacologically relevant levels in *A. pinnata* pith. Accordingly, the selection of these compounds for network pharmacology and molecular docking should be regarded as a hypothesis-generating extension rather than direct evidence that they are the principal active constituents of the pith. Targeted LC-MS/MS analysis using authentic standards will be required to confirm their chemical identities and quantify their abundance (Wang et al., 2022).

Third, network pharmacology, PPI analysis, and molecular docking provide predicted compound–target relationships but cannot establish direct biological interactions or therapeutic efficacy. The high connectivity of AKT1, MMP9, PTGS2, PPARG, and other proteins reflects their topological importance within database-derived interaction networks rather than confirming that they are definitive therapeutic targets of *A. pinnata*-derived compounds. Likewise, docking scores indicate predicted structural compatibility and should not be interpreted as experimentally confirmed binding. Future studies should therefore evaluate these candidate targets using direct binding assays, enzyme-activity measurements, gene perturbation, chondrocyte-based experiments, and appropriate animal models.

Finally, the machine-learning analysis was based on a relatively small, single human OA dataset. Although DDIT3, KLF10, CFI, and MAFF showed strong internal discriminatory performance, these results may be affected by sample size, model overfitting, and dataset-specific characteristics. Independent external cohorts and rigorously separated training and validation datasets will therefore be necessary before these genes can be considered clinically useful biomarkers.

Overall, the present study should be regarded as a comparative plant-omics investigation accompanied by exploratory computational analyses that generate testable hypotheses for future pharmacological studies.

## 5 Conclusion

This study demonstrates substantial geographical variation in the metabolic and transcriptional profiles of *A. pinnata* pith collected from five geographical origins. The observed differentiation was particularly associated with flavonoid and phenylpropanoid metabolism, together with amino-acid, lipid, central-carbon, and glutathione-related pathways, indicating that geographical variation in *A. pinnata* pith involves coordinated changes in both secondary and primary metabolic processes. These findings provide a comparative molecular framework for evaluating geographical resources and chemical variation in *A. pinnata*.

The OA-related analyses should be interpreted separately from the experimentally observed geographical variation. Network pharmacology and molecular docking generated predicted compound–target relationships for quercetin and naringenin, whereas machine-learning analysis of the independent human OA dataset identified DDIT3, KLF10, CFI, and MAFF as candidate OA-associated genes. These findings are computational and hypothesis-generating and do not demonstrate that *A. pinnata* pith has anti-OA activity or that the predicted molecular interactions occur under biological conditions.

Further studies should therefore confirm the chemical identities and abundance of the relevant flavonoid compounds using targeted quantitative methods and subsequently evaluate their biological activities through direct binding assays, cellular models, gene-intervention experiments, and appropriate animal studies. Overall, the present study establishes a molecular basis for understanding geographical variation in *A. pinnata* pith and provides experimentally testable hypotheses for future investigation of selected bioactive constituents.

## Data Availability

The original contributions presented in the study are included in the article/**Supplementary Material**. Further inquiries can be directed to the corresponding authors.
